# Reduced keratin expression in colorectal neoplasia and associated fields is reversible by diet and resection

**DOI:** 10.1136/bmjgast-2014-000022

**Published:** 2015-04-17

**Authors:** Caroline A Evans, Ria Rosser, Jennifer S Waby, Josselin Noirel, Daphne Lai, Phillip C Wright, Elizabeth A Williams, Stuart A Riley, Jonathan P Bury, Bernard M Corfe

**Affiliations:** 1 Department of Chemical and Biological Engineering, ChELSI Institute, University of Sheffield, Sheffield, UK; 2 Molecular Gastroenterology Research Group, Department of Oncology, University of Sheffield, The Medical School, Sheffield, UK; 3 Department of Biological Sciences, The University of Hull, Hull, UK; 4 Conservatoire National des Arts et Mmétiers, Paris, France; 5 Department of Geography, University of Sheffield, Sheffield, UK; 6 Human Nutrition Unit, Department of Oncology, University of Sheffield, The Medical School, Sheffield, UK; 7 Department of Gastroenterology, Northern General Hospital, Sheffield, UK; 8 Department of Pathology, Royal Hallamshire Hospital, Sheffield, UK; 9 Insigneo Institute for in Silico Medicine, The University of Sheffield, Sheffield, UK

**Keywords:** ADENOMA, BUTYRATE, CYTOKERATINS, DIETARY FIBRE

## Abstract

**Background:**

Patients with adenomatous colonic polyps are at increased risk of developing further polyps suggesting field-wide alterations in cancer predisposition. The current study aimed to identify molecular alterations in the normal mucosa in the proximity of adenomatous polyps and to assess the modulating effect of butyrate, a chemopreventive compound produced by fermentation of dietary residues.

**Methods:**

A cross-sectional study was undertaken in patients with adenomatous polyps: biopsy samples were taken from the adenoma, and from macroscopically normal mucosa on the contralateral wall to the adenoma and from the mid-sigmoid colon. In normal subjects biopsies were taken from the mid-sigmoid colon. Biopsies were frozen for proteomic analysis or formalin-fixed for immunohistochemistry. Proteomic analysis was undertaken using iTRAQ workflows followed by bioinformatics analyses. A second dietary fibre intervention study arm used the same endpoints and sampling strategy at the beginning and end of a high-fibre intervention.

**Results:**

Key findings were that keratins 8, 18 and 19 were reduced in expression level with progressive proximity to the lesion. Lesional tissue exhibited multiple K8 immunoreactive bands and overall reduced levels of keratin. Biopsies from normal subjects with low faecal butyrate also showed depressed keratin expression. Resection of the lesion and elevation of dietary fibre intake both appeared to restore keratin expression level.

**Conclusion:**

Changes in keratin expression associate with progression towards neoplasia, but remain modifiable risk factors. Dietary strategies may improve secondary chemoprevention.

**Trial registration number:**

ISRCTN90852168.

**Summary box**

**What is already known about this subject?**

▸  Factors affecting adenomagenesis and carcinogenesis are thought to be different. Previous work has shown that the relative risks of each vary between populations. Patients with a history of adenoma are at greater risk of metachronous adenoma, which may support the existence of colorectal fields. To date, little is known about the molecular basis for such fields.
▸  Keratins are a type of intermediate filament proteins, which as part of cellular cytoskeleton have important regulatory functions on the colonic mucosa. K8 null mice develop colitis and K8 is shown to modulate tumour necrosis factor (TNF) action.
▸  Butyrate is widely hypothesised as being a key chemoprotective molecule, through effects on cellular programming and cell fate determination and more recently through regulation of metabolic phenotype of the cell.

**What are the new findings?**

▸  Validated proteomic analysis of the macroscopically normal mucosa in the colon of participants with an adenoma was compared to tissue from the adenoma and from participants free of pathology. The analysis indicated that several groups of proteins were altered, but particularly proteins of the keratin family.
▸  These data suggest that there may be field-wide changes in the colon associating with the presence of a lesion, and contribute to the molecular evidence for existence of fields.
▸  These changes may be restored to a normal phenotype (for keratins) by resection of lesions and by dietary fibre interventions.

**How might it impact on clinical practice in the foreseeable future?**

▸  The data provide evidence for the existence of colorectal fields. As such this introduces the possibility of using markers (eg, the levels, distributions and form of keratin) as biomarkers of fields. Critically this work suggests that active interventions may modulate fields, implying they are regressible. Both resection and increased intake of fibre rich food both restored normality to keratin expression. As such secondary chemopreventive strategies have been proven in principle and offer the opportunity to give lifestyle advice to modulate risk of metachronous disease.

## Introduction

Metachronous adenoma (AD) is a greater risk than incident AD,^1^ an observation that has been used in support of a theory of colonic field effects. The existence of field effects around lesions or giving rise to lesions was proposed over 50 years ago.^2^ In the case of the colon, there is debate on whether the immediate region around the lesion or the entire colon represents the field. We have recently summarised the evidence and proposed a series of qualitative models for metachronous AD.^3^ Direct molecular evidence of field effects in the colon has remained elusive but a gel-based proteomic analysis by Polley *et al*^4^ identified proteins altered in the vicinity of ADs, including keratin 8. This study supported fields being local to the lesion, but did not examine sites distant to the lesion. Additional indirect evidence for crypt expansion to fields comes from measuring extent of hyperploidy and crypt expansion in patients with colitis.^5^ Nonetheless, direct evidence for molecular changes in colorectal fields remains limited.

Keratins are key components of intermediate filaments, a cytoskeletal structure responsible for the structural integrity of the epithelium through cell-cell contacts, cell shear stress and through regulation of signal transduction and cell polarisation. Keratin expression occurs in specific combinations, dependent on the type of epithelium, and differentiation status related to the epithelial type and stage of cellular differentiation.^6^ K8, K18 and K19 are the predominantly expressed in colonic epithelium.^7^ Keratin 8 (K8) forms a heterodimer with keratin 18 (K18) in the colonocyte. Several lines of evidence suggest a role for K8 in maintenance of a functional colorectal epithelium. Alteration in K8 patterns occur around colorectal neoplasia^4^; polymerisation-inhibiting mutations in K8 were observed in a subset of inflammatory bowel disease (IBD) patients^8^; transgenic mice lacking K8 exhibit hyperplasia in the colon and impaired barrier and absorptive function in addition to developing colorectal neoplasia.^9^ The literature on associations between keratin expression, function and colorectal disease has recently been reviewed.^7^

Keratins (and other proteins in cancer pathways) are subject to modification by acetylation.^10^ In the case of keratin we have shown that acetylation may drive transition between different protein states (soluble/insoluble).^11^ ^12^ There is substantial evidence from epidemiological studies to suggest that high dietary fibre intakes may protect against colorectal cancer.^13^ ^14^ Mechanisms may include reduction in transit time, increase in stool volume, both of which would reduce exposure to carcinogens, and butyrate production. Butyrate, produced by bacteria-mediated fermentation regulates cell cycle and apoptosis in vitro*,*^15–17^ and is chemopreventive in rat models of colorectal carcinogenesis.^18^ These properties are attributed to butyrate's molecular action as an inhibitor of histone deacetylation which results in increased protein acetylation.^19^ Several microarray-based studies^20^ ^21^ reveal a substantial portion of the transcriptome is altered in response to butyrate but that protein acetylation is not limited to histones and acetylation is as important in the regulation of protein function as phosphorylation.^22^

Proteomic approaches to the analysis of clinical specimens allow objective and impartial evaluation of the changes occurring between compared samples. Proteomics is a suite of protein separation and characterisation approaches, employing gel and gel-free approaches for protein separation. Two dimensional gel electrophoresis (2DGE) separates proteins by charge and mass, gel-free approaches such as isobaric tags for relative and absolute quantification (iTRAQ) enable quantitative and global profiling in a multiplex format,^23^ ^24^ with identification of larger numbers of proteins, and the relative quantification of all identifiable proteins, but with the limitation of loss of separation of subspecies of a protein, for example distinction of a phosphorylated and non-phosphorylated species. iTRAQ is an emerging standard for assessment of changes in a global proteome, while 2DGE has utility for assessment of post-translational modifications of smaller numbers of species.^10^

We applied multiple proteomic workflows to protein extracts from biopsies taken at, near and distant to colorectal neoplasia, stratified by levels of butyrate present in a stool sample to explore the effects of both colorectal fields and of butyrate on the status of epithelial keratins.

## Methods and materials

### Participants and recruitment

This study includes two arms: a cross-sectional study and a fibre-intervention. The study design has been reported elsewhere.^25^ Ethics committee approval was obtained from the North Sheffield Research Ethics Committee prior to recruiting (Reference number: 06/Q2308/93).

#### Cross-sectional study (FACT OBS)

Participants included in this study were recruited from colonoscopy lists at Sheffield Teaching Hospitals between October 2007 and June 2008. In total 62 participants were included in this analysis, of whom 34 were found to be free from disease during endoscopy (the normal group), while 28 participants were found to have histologically confirmed ADs (the AD group). The normal group had a younger mean age (62.1±11.4 vs 68.1±0.1, p=0.047). There were no significant differences between groups for BMI or weight. In patients with adenomatous polyps biopsies were taken from the AD itself, and from macroscopically normal mucosa on the contralateral (CL) wall to the adenoma CL and from the mid-sigmoid (MS) colon (see online supplementary information (SOI); section 1). Endoscopists judged on macroscopic appearance which polyps were adenomatous, all judgements were independently confirmed by a histopathologist and non-adenomatous samples were removed from the analysis. In lesion-free participants biopsies were taken from the MS. Three biopsies were taken at each site—two for proteomic analysis and one for immunohistochemistry. Participants additionally provided a stool sample (while bowel habit was normal) for assessment of faecal SCFA levels.

#### Intervention study (FACT INT)

An initial screening tool for fibre intake was developed (DL and EAW, unpublished) to identify participants with low dietary fibre intake (<12 g/day). The screening tool was in the form of a brief food frequency questionnaire that asked about the consumption and portion size of high fibre breads, (cereals, pasta, rice, vegetables and fruit). Nine participants were included in the intervention trial. Biopsies were taken during scheduled endoscopy from MS only and either flash frozen for protein analysis or formalin fixed for IHC. Diet at baseline was assessed using a 4-day food diary (FD) which confirmed low baseline fibre intake. A food replacement strategy was used to increase fibre intake. The high fibre intervention was 8 weeks in duration and included an initial 2-week period when intake was increased incrementally. Increased fibre consumption was encouraged via supply and weekly delivery of fibre-rich foods (cereals, breads, fruit, vegetables) directly to the participants’ homes. Participants were invited to choose their own foods from a list of high-fibre foods and were offered advice as to how to incorporate more fibre into their diets. Participants were provided with a simple fibre reckoner to assess their own daily fibre intake and were asked progressively to increase fibre intake, to achieve an equivalent of over 20 g/day by the end week of the intervention. FD, endoscopy, biopsy and stool sampling were repeated at the end of the intervention.

### Biopsy lysis, pooling and fractionation

**Figure 1:**
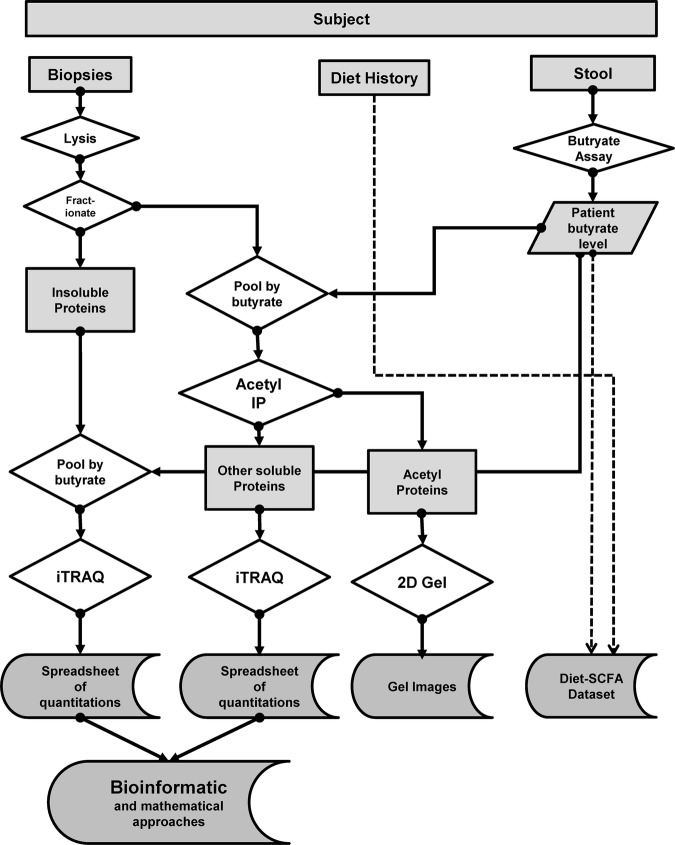
Experimental workflow. The figure summarises the workflow used in this study. Participants recruited had biopsies taken and provided stool (for faecal butyrate) and diet information. The frozen biopsies were lysed to yield soluble and insoluble fractions. The former was pooled according to butyrate and subject to immunoprecipitation for acetyl proteins, to yield an acetyl-enriched and a soluble fraction. The former was analysed by 2DGE whereas the latter was analysed by iTRAQ. The insoluble fraction was processed to yield IF and then analysed by iTRAQ. iTRAQ, isobaric tags for relative and absolute quantification; 2DGE, two dimensional gel electrophoresis; IF, intermediate filament.

Colorectal pinch biopsies (∼5 mg) were suspended in kinase buffer (50 mM Tris-HCl pH7.5; 10 mM MgCl_2_; 0.1 mM EDTA; 2 mM DTT) followed by homogenisation. The lysate was centrifuged to provide crude separation between the insoluble and soluble fractions. Resulting fractions were grouped by diagnosis and region and were ranked by faecal butyrate level and then pooled: eight lysates (two each from four patients) were used in each pool. Acetyl proteins were separated from other soluble proteins using antibody immobilised to a Pierce Seize matrix. The insoluble fraction, principally intermediate filaments, was prepared using our integrated workflow for iTRAQ-compatible analysis^26^ which modified the high-salt extraction technique of Achstaetter^27^ and Herrmann.^28^ The insoluble and soluble fractions were analysed using iTRAQ workflows while acetyl proteins were analysed by 2D gel electrophoresis (*vide infra*). The workflow is summarised in figure 1.

### Proteomic methods

*iTRAQ workflow* Proteomes were analysed using an iTRAQ workflow as previously described.^29^ Briefly proteins were reduced, alkylated and subject to proteolytic digestion using trypsin prior to labelling using the iTRAQ 8 plex kit (ABSciex, Warrington, UK), according to manufacturer's instructions. Peptide fractionation was undertaken using Strong Cation Exchange on a BioLC HPLC system (Dionex, Surrey, UK). Fractions were collected, dried and stored prior for mass spectrometric analysis. Mass spectrometry (MS) was performed using ESI-qQ-TOF-MS/MS platforms (Bruker, Bremen, GmBH) coupled with an online nano-flow liquid chromatography system (U3000, Dionex, Camberley, UK). Protein identifications were obtained using the the Phenyx software platform (GeneBio, Geneva) to perform database searching against the human UniProt (SwissProt, Trembl downloaded 11 May 2010) database. A concatenated target-decoy database search strategy was also employed to estimate the rate of false discovery rate,^30^ calculated to be 1% and as such were well within the 5% recommended for reporting proteomic data.^31^

### Mathematical, bioinformatic and statistical analysis

Hierarchical clustering and principal component analysis were performed to group the data based on the degree of similarity between samples. Agglomerative clustering using the squared Euclidean distance between log_10_ iTRAQ ratios and smallest inter-cluster dissimilarity linkage procedure was performed (Mathematica 7.0.0 for Mac). Statistically significant changes in protein level were identified using our t test algorithm.^32^ Pathway analysis was undertaken using the Instance Browser in Reactome.^33^ Protein interaction networks were analysed using STRING V. 9.0.^34^ Statistical analysis of immunoblot densitometry data and immunohistochemical data was carried out in Microsoft Excel and SPSS.

### Immunoblotting

Proteins were separated by SDS-PAGE and transferred to PVDF. Membranes were incubated with primary antibody solutions in blocking buffer. Primary antibodies used include: keratin 19 (mAb3238, Millipore, UK); ApoA1(Ab48647, Abcam, UK), M2PK (Ab38327, Abcam, UK); GAPDH (AM4300, Ambion); and α-tubulin (Ab7792, Abcam). Cross-reactions were visualised using HRP-conjugated secondary antibodies (Dako), Immobilon Western HRP substrate (Millipore, UK). A Chemigenius Bioimaging system was employed for band visualisation and densitometric analysis.

### Immunohistochemistry for keratin 8

Our previously established protocols for keratin 8 immunohistochemistry and scoring were used.^35^ Antigen retrieval was performed on 4-micron formalin-fixed, paraffin-embedded sections with EDTA (1 mM, pH8) in microwave at high power for 8 min. Sections were incubated with primary antibody (K8 mouse monoclonal, ab9023) and biotinylated secondary antibody (anti mouse IgG, RTU Vectastain Universal). Detection was performed using DAB kit (sk-4100Vector lab, UK).

## Results

### Recruitment and participant demographics

The inclusion, exclusion criteria, justification and study design have previously been reported.^36^ Of 81 male participants attending for diagnostic colonoscopy were recruited, 19 were excluded from pooling by butyrate owing to a diagnosis of cancer (n=11), failure to provide a stool sample (n=3), or bowel preparation other than Kleanprep (n=5). Biopsies from participants prepared for endoscopy with Picolax (n=5) were pooled separately as bowel preparation influences proliferation of colon epithelial cells.^37^ Colonoscopy was completed to the caecal pole in all participants. Adenomatous polyps (confirmed histologically) were present in 28 participants and macroscopically normal colonic mucosa in the remaining 34. The morphometric and AD positional data are shown in the SOI, section 4.

Pooling for proteomics was undertaken following stratification by faecal butyrate concentration. Stool samples were extracted and butyrate determined as previously described.^35^ ^38^ There was no significant difference in the normal and AD subject groups’ highest (15.5 mM vs 13.0 mM) and lowest (0.9 vs 0.8 mM) mean butyrate levels (SOI, section 4). Individuals were stratified by faecal butyrate, and samples grouped by diagnosis/biopsy location (normal MS, AD MS, CL and lesion AD).

### Workflow

The workflow for this study is set out in figure 1. The insoluble fraction was processed to yield intermediate filament (IF)-enriched material.^26^ The soluble fractions were pooled in fours (empirically determined to yield analysable acetyl-proteins by IP, data not shown). Samples from the highest and lowest butyrate pools from normal participants, and AD participants at each of the three biopsy sites (a total of eight pools) were immunoprecipitated to yield a pooled IP eluate (acetyl proteins), and a pooled flow-through (other soluble proteins).

For proteomic analysis, the insoluble and soluble pools were analysed by 8-plex iTRAQ workflow; the IP pools were analysed by 2DGE. IP pools were indicative of a profound effect of bowel preparation on protein modification (SOI, section 15) and this analysis was not pursued further. Orthogonal validation was provided at two tiers: (1) immunoblot analysis of the pooled protein extracts analysed by iTRAQ and (2) IHC on archived FFPE biopsy material for individual participants from the study.

### The insoluble proteome shows lesion-associated changes in the keratin profile

**Figure 2:**
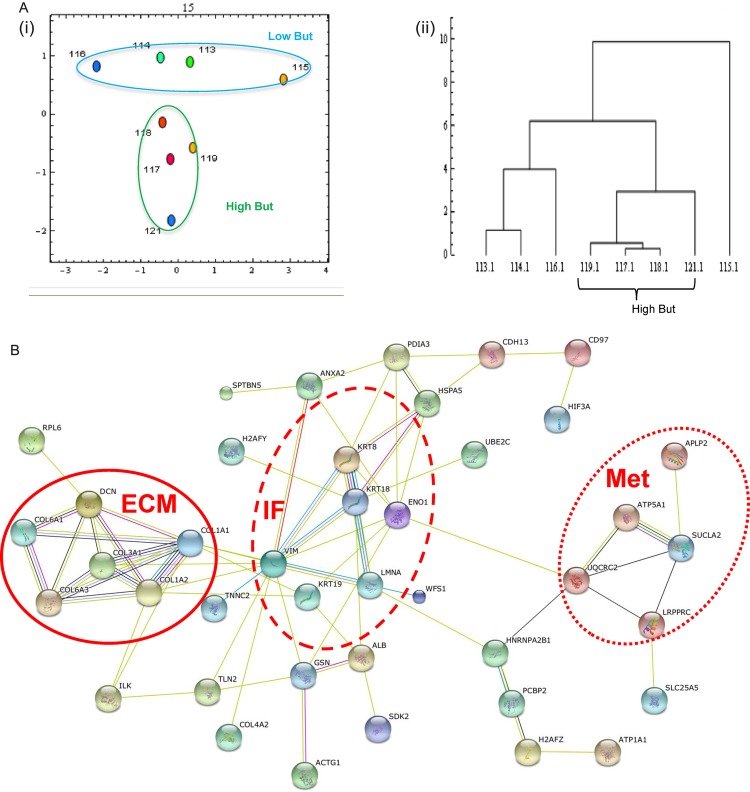
iTRAQ analysis of the insoluble proteome shows effects of lesional proximity and butyrate level. Samples were separated and relatively quantified by 8-plex iTRAQ. Global analysis of the data was undertaken by principal component analysis (PCA) (panel A) and hierarchical clustering analysis (HCA) (panel B). PCA showed clustering by butyrate level with low butyrate samples in the blue oval and high butyrate in the orange oval. HCA showed the high-butyrate samples were more alike than the other samples. A protein interaction network was generated from the whole dataset (orphan nodes not shown) indicating proteins interlinked with clusters around extracellular matrix (solid line), keratins (dashed line) and metabolism (dotted line). Significant differences between samples according to lesional proximity and controlling for butyrate were computed and are shown in panel C. Proteins listed in red are significantly downregulated, while those in green are upregulated. iTRAQ, isobaric tags for relative and absolute quantification.

A small number of studies have converged on keratins as hallmarks of colorectal mucosal health^7^ we undertook a proteomic analysis of the insoluble fraction which is enriched for intermediate filament proteins, including keratins.^26^ Samples grouped by diagnosis/biopsy location (normal MS, AD MS, CL and lesion AD) and were stratified by low (∼1mM) and high (∼14 mM) butyrate (see above and SOI for concentrations). The iTRAQ workflow identified 55 proteins were represented by ≥2 peptides (complete list: SOI, section 5). Intermediate filament proteins, including keratins were present. Principal component analysis (PCA) (figure 2Ai) of the proteins and associated iTRAQ ratios across the data set, indicated that the samples clustered clearly according to butyrate status, with a single axis (component 2) distinguishing low from high butyrate. The data were further analysed using a Euclidian agglomerative clustering approach, the data suggested that the high-butyrate group were more alike than the low-butyrate group. Interacting protein networks were identified with STRINGS DB (figure 2B and SOI, section 6). Intriguingly, when all lines of evidence are considered the entire intermediate filament proteome forms a single interaction network, with clustering around the keratin-vimentin and collagen centres in additional to a metabolism cluster. A pathways analysis using Reactome (SOI, section 7), indicated that collagen formation and extracellular matrix organisation were well represented (p=2.8×10^−8^ and 1.8×10^−8^, respectively). The pathway for NCAM signalling in neurite outgrowth was very highly represented (p=1.2×10^−10^). The data are consistent with the proposal that IF networks form scaffolding on which associated proteins organise and are regulated to control metabolic activities.^39^

We hypothesised that the alterations in insoluble keratins observed associated with lesions or butyrate level could either reflect a reduction in total keratin in the tissue, or could represent a shift in protein space into the soluble phase. We therefore undertook undirected and directed analyses of the soluble fractions to establish whether changes in keratin were reflected across the fractions (*vide infra*).

Parallel analysis of the soluble fraction reveals observed changes in keratin are at protein level, not a change in protein solubility.

**Figure 3:**
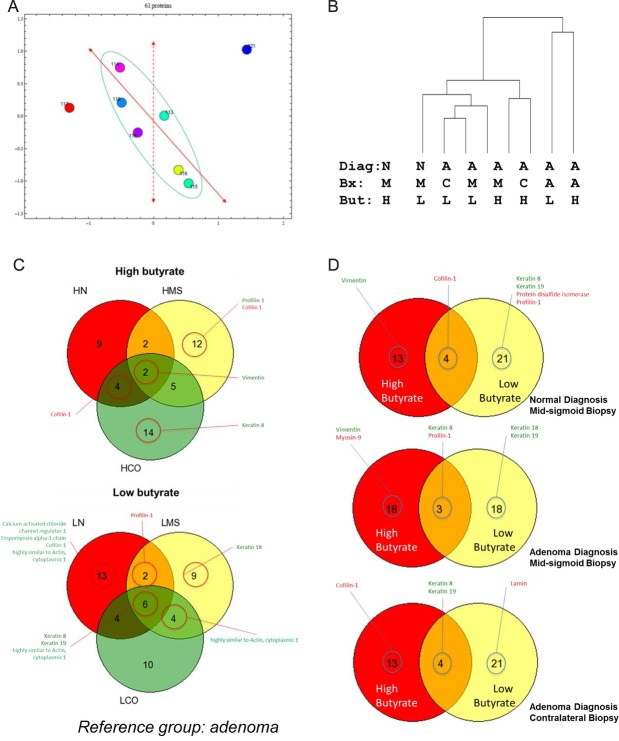
iTRAQ analysis of the global soluble proteome shows effects of butyrate and presence of an adenoma. Samples were separated and relatively quantified by 8-plex iTRAQ. Global analysis of the data was undertaken by principal component analysis (PCA) (panel Ai) and hierarchical clustering analysis (HCA) (panel Aii). PCA showed macroscopically normal samples could be separated by a single factor to distinguish high from low butyrate (dashed axis) and a straight line (function of two factors) could separate normal from lesion-associated samples. Panel B hierarchical clustering was used to group the data based on the degree of similarity between the samples analysed using the complete iTRAQ data set. Quantitative data were used to identify significant differences between the soluble proteome samples according to lesional proximity and controlling for butyrate. Panel C shows comparisons of lesion proximity in high and low butyrate samples and panel D shows changes associated with butyrate level, controlling for lesional proximity. Proteins listed in red are significantly down-regulated, while those in green are up-regulated. iTRAQ, isobaric tags for relative and absolute quantification.

SDS PAGE analysis of the soluble fraction indicated that the proteins remained intact without degradation or smearing and that no part of the mass range was distorted (SOI section 8). The soluble proteome was analysed by iTRAQ (SOI section 9). Analysis using Instance Browser showed that no pathways were particularly enriched (SOI, section 10). PCA (figure 3Ai) shows that the macroscopically normal tissues are much more similar than the lesional tissue. These samples may be further separated along linear axes. The dotted axis suggests that one component alone can distinguish the effects of butyrate on tissue. The solid axis demonstrates separability of macroscopically normal tissue according to the presence or absence of a lesion. Hierarchical clustering analysis (HCA) suggested again that lesional tissue is most divergent, but that within macroscopically normal tissue, the effect of butyrate is greater than the effect of lesional proximity (figure 3Aii). Protein interaction networks were built as described above. A large network accounted for many of the proteins, with several smaller clusters (SOI section 11). Significant changes were analysed either by controlling for the butyrate status and assessing the impact of lesion on the proteome (figure 3C) or by controlling for the lesional status and assessing the impact of butyrate on the proteome (figure 3D). For clarity on cytoskeletal and related proteins are shown, versions with complete listings appear in the SOI (SOI, section 11). Venns show proteins significantly altered at each site by high or low butyrate.

Seven proteins were subject to orthogonal validation by western immunoblot: keratin 8, keratin 19, α-tubulin, ApoA1, M2PK, GAPDH and cofilin (SOI section 12). The data show different responses to butyrate with progressive lesional proximity. Assessment of the effect of lesional proximity on ApoA1, tubulin and keratins 8 and 19 was undertaken (SOI, section 13). ApoA1 and α–tubulin show a consistent trend of increased expression with transition from normal to field to AD tissue (SOI, section 13). In contrast keratins 8 and 19 show a consistent trend towards downregulation across the same series.

**Figure 4:**
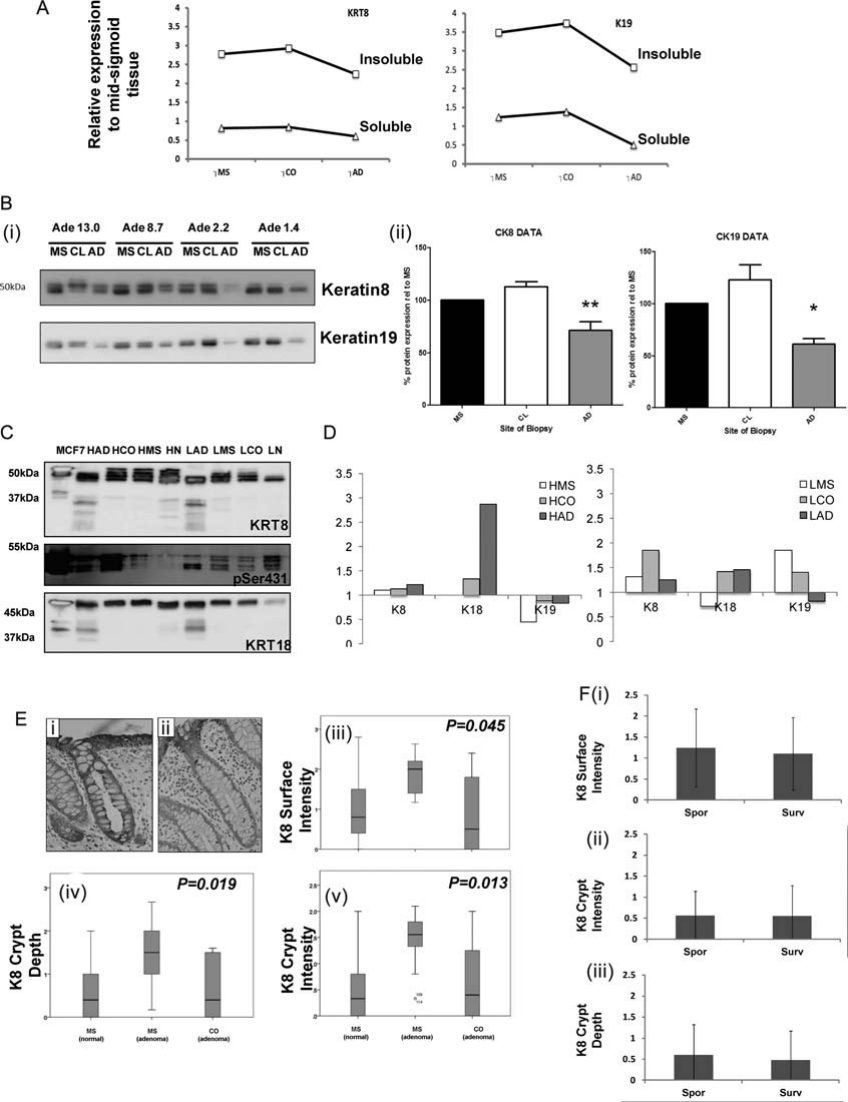
Integrative analysis of changes in keratin. Data on relative expression of keratins 8, 18 and 19 are extracted from the isobaric tags for relative and absolute quantification (iTRAQ) analyses and presented in panel A to allow comparison of trends across samples. Panel Bi shows immunoblot for keratin 8 and keratin 19 of different soluble fraction pools with varying mean butyrate at the mid-sigmoid (MS), contralateral (CL) and adenoma (AD) biopsy sites. Bands were quantified by densitometry and are represented in Bii, MS is used as the reference sample, levels at the CL wall and lesion AD are shown in the white and grey bars respectively. Panel C shows immunoblot analysis of keratin 8 and 18 immunoreactivity in the insoluble fractions at varying butyrate level (high and low). Comparative analysis of trend in change in the iTRAQ data for soluble and insoluble fractions is shown in Panel D. FFPE sections were stained and scored for keratin 8. Panels Ei and Eii show representative sections for high and how scores. Three aspects of keratin organisation were scored: surface intensity, crypt intensity and crypt depth. Box-and-whiskers plots show distributions of each data for each end point between three different sample sets—mid-sigmoid (normal and adenoma) and contralateral to adenoma. All end points the data showed significant differences (Jonkheere-Terpstra). When the normal group were separated into new cases (sporadic—spor) or surveillance cases free from pathology but with a history of adenoma (surveillance) there were no significant differences between end points.

Data for keratin were extracted each iTRAQ to allow comparison of the changes observed between the soluble and insoluble fractions in the same the same subject pools were undertaken. Analysis revealed consistent trends in data (figure 4A), supporting a model whereby changes in keratin reflected the change in the total level of protein in association with a lesion or levels of butyrate. Further orthogonal validation by antibody-based methods (western immunoblotting, immunohistochemistry) was undertaken (figure 4B–F). Soluble fraction pools were analysed by western immunoblot (figure 4Bi) for keratin 8 and keratin 19. Quantification revealed that their level is constant between the MS and CL sampling positions, but that in lesions levels are significantly reduced (figure 4Bii). In the insoluble fraction, analysis of the proteomic data (figure 4C) indicated that alteration in keratin 8 and 18 was pronounced around lesions and that this was also a function of butyrate status. Immunoblot of keratin 8 and 18 in insoluble samples (figure 4C) shows that the keratin 8 immunoreactivity profile was markedly different to the soluble form: in the insoluble material the keratin 8 pool was represented by multiple bands in the 50 kDa region. Samples extracted from lesional tissue had lost some of the higher molecular weight forms, and lower bands appeared, suggestive of proteolysis. The immunoreactivity profile of low butyrate samples from macroscopically normal tissue resembled the lesional tissue, with loss of the higher molecular weight forms of keratin. The immunoblots suggest reduction is greater in low butyrate conditions.

**Figure 5:**
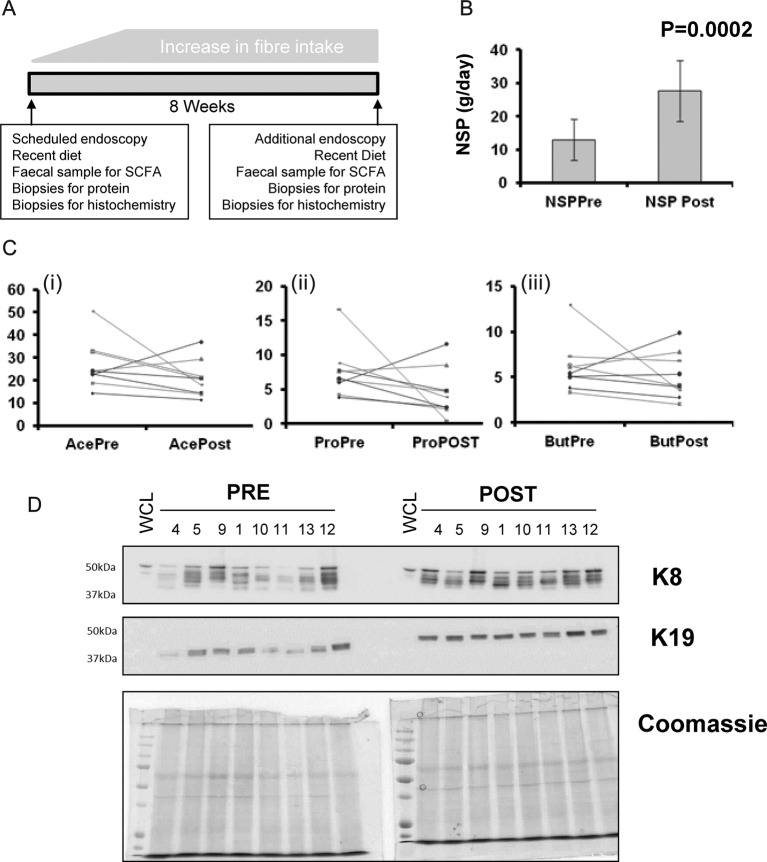
A high-fibre intervention elevates keratin level. Panel A graphical protocol: participants were recruited to an 8-week fibre intervention. Biopsies, food diary and faecal samples were collected at baseline and at exit from the intervention. Non-starch polysaccharide intake was significantly increased (panel B), however, there were no significant effects on faecal acetate or butyrate (Ci, Ciii) whereas propionate (Cii) approached a significant reduction. Levels of keratins 8 and 19 were measured by immunoblot, in samples preintervention and postintervention. Owing to effects of SCFAs and diet on ‘housekeeping’ markers (not shown), Coomassie staining of duplicate gels was used to show controlled loading of gels.

Since the trend is the same in the soluble and insoluble fractions (using iTRAQ and western immunoblot analysis) the hypothesis of change in cellular level of each of K8, K19 is a better fit to the data. To assess whether changes were reflected at a histological level and to allow analysis at individual subject level in unpooled samples, FFPE sections were assessed using our protocol.^35^ Figure 4Ei and ii show examples of crypts with strong and deep staining and weak and shallow staining, respectively, reflecting the scoring criteria developed: crypt depth (KCD), crypt intensity (KCI) and surface intensity (KSI) (*ibid*.). Analysis in figure 4Eiii, iv and v shows each end point against lesional proximity in samples from MS (normal) MS (AD present) and CL to AD. All outcomes were tested statistically and revealed significant differences between each measure and lesional proximity (p=0.019, p=0.014, p=0.045, respectively, Jonkheere-Terpstra test). As the normal group was a mixture of individuals from the AD surveillance and index colonoscopies, retrospective data on history of AD was obtained (where available) and a subgroup analysis undertaken. There were no significant differences between any of the keratin end points between subgroups (figure 5Fi-iii and SOI, section 14). Taken together these data reveal significant differences in expression of multiple keratins and their products as a function of butyrate status and lesional proximity. The similarity between the sporadic and surveillance subgroups suggests that the changes observed associated with presence of a lesion may be reversible by lesional resection.

### A fibre intervention elevates keratin expression

As dietary fibre intake is associated with a reduced risk of cancer,^13^ we assessed the effect of increasing fibre intake on keratin expression. A visual protocol is shown in figure 5A, and details are in the methods and reference.^24^ Mean fibre intake was very significantly increased (figure 5B), although surprisingly little effect was seen on levels of faecal acetate or butyrate (figure 5Ci and iii), and a trend towards reduced propionate was noted (p=0.086, figure 5Cii). Soluble protein fractions from these biopsies were extracted and immunoprobed for K8 and K19 (figure 5D). Expression of both proteins was higher following fibre intervention.

## Discussion

In this study we have collected biopsies from participants with and without a neoplasia at positions with various proximities to the lesion. These were subject to a three-tier proteomic analysis, with orthogonal validation by immunoblot and immunohistochemistry and finally an independent intervention trial. Our experimental design reveals a very consistent pattern of decreased global level of keratins in lesional tissue and with butyrate concentration. Polymerised keratins are a key component of IF, and occupy the insoluble fraction of lysed material, whereas depolymerised forms may appear in the soluble fraction. The consistent pattern of change between these fractions is indicative of reduced global level of keratin with lesional proximity. The protein acetylation environment of epithelia will be influenced by microenvironmental levels of butyrate.^40^ ^41^ We and others have also shown that keratins are themselves acetylated^10^ ^42^ and that keratin acetylation is associated with depolymerisation.^11^ ^12^ Our data also indicate that keratin forms may be altered as a consequence of the lesion and butyrate status: in lesional tissue the insoluble keratins appeared to be degraded, an observation paralleled in low-butyrate conditions in lesion-free participants. This suggests a contributing chemoprevention action of butyrate. Critically, keratin changes may remain modifiable risk factors: the fibre intervention increased keratin level and retrospective subgroup analysis implied restoration of keratin postpolypectomy.

Changes in metabolic proteins were also noted. The colonocyte utilises butyrate as a fuel source,^43^ proximity to a lesion impacts on the expression profile of metabolic enzymes, possibly indicating local areas of Warburg metabolism in a primarily β–oxidative tissue. Alterations in pyruvate handling may change the functional threshold for butyrate's histone deacetylase (HDAC) inhibitory activity.^41^

### An integrative model for pre-neoplastic changes

**Figure 6:**
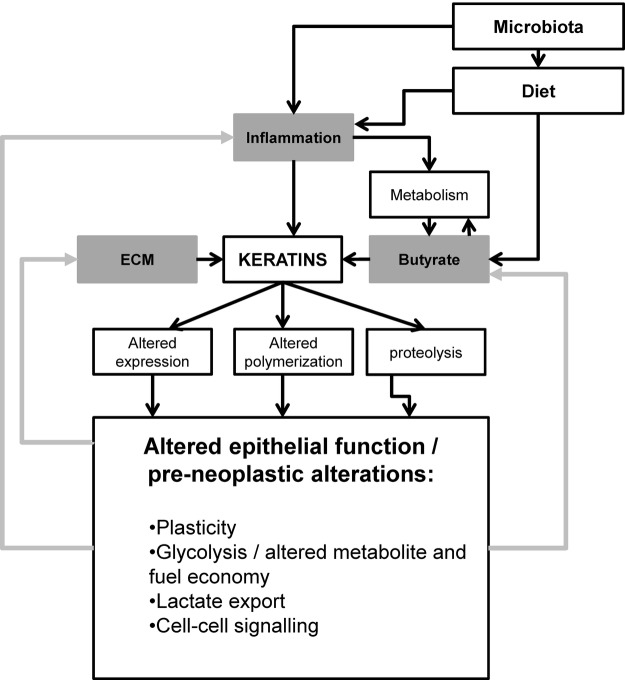
Integrative model of impact of microenvironment on keratin and functional consequences.

Figure 6 shows an integrative model accounting for parameters we report in the context of the wider literature. Keratins are responsive to butyrate resulting in changed expression and polymerisation, however, we have also recently reported upregulation of keratin 8 in response to inflammation in active colitis (Corfe *et al* submitted to *Journal of Pathology*). Subclinical inflammation is a risk factor for carcinogenesis and has recently been shown to impact on fatty acid oxidation.^44^ The extracellular matrix, contributes to cell viability signalling, and alters flexibility in the neoplastic colon, interactions with and may signal via keratins. As such the metabolic, inflammatory and extracellular matrix environments may all impact on keratin at the levels of expression, polymerisation and degradation. The inflammatory and metabolic environments are partly a function of diet and the microbiome: diet may directly influence inflammation (eg, through imbalance of ω-3 and ω-6 inter alia), and carbohydrate and fibre and the microbiome together mediate the levels of SCFA in the gut. We propose a model wherein modifiable risk factors may exert subclinical effects on the colonocyte, exhibited or integrated through changes in keratin and intermediate filament function. In turn such changes may form a positive feedback loop, accelerating dysbiosis or inflammation.

Our data provide direct molecular evidence of field-changes. In the preneoplastic colon these events remain, in principle, modifiable and offer a therapeutic window. Future research should address whether such changes are cause or effect of neoplasm.

## Supplementary Material

Supplementary Materials
