# Supplementary material for: Reduced keratin expression in colorectal neoplasia and associated fields is reversible by diet and resection
Source: BMJ Open Gastroenterol. 2015 Apr 17;2(1):e000022. doi: 10.1136/bmjgast-2014-000022 (PMC4599164; doi:10.1136/bmjgast-2014-000022)
Supplement: Supplementary Materials [file bmjgast-2014-000022.html]

Reduced keratin expression in colorectal neoplasia and associated fields is reversible by diet and resection: BMJ Open Gastroenterology: Vol 0, No 0

Online Supplement
