# Supplementary material for: Reduced keratin expression in colorectal neoplasia and associated fields is reversible by diet and resection
Source: BMJ Open Gastroenterol. 2015 Apr 17;2(1):e000022. doi: 10.1136/bmjgast-2014-000022 (PMC4599164; doi:10.1136/bmjgast-2014-000022)
Supplement: Supplementary Materials [file bmjgast-2014-000022supp.pdf]

Online Data Supplement

for:

Reduced keratin expression in colorectal neoplasia and associated fields is reversible by both diet and resection

Caroline A Evans, Ria Rosser, Jennifer S Waby, Josselin Noirel, Daphne Lai, Phillip C Wright, Elizabeth A Williams. Stuart A Riley, Jonathan P Bury & Bernard M Corfe

Author to whom correspondence should be addressed: Dr Bernard Corfe, Molecular Gastroenterology Research Group, Academic Unit of Surgical Oncology, Department of Oncology, The Medical School, Beech Hill Road, Sheffield, S10 2RX,

email: b.m.corfe@shef.ac.uk ,

Tel: +44 (0) 114 271 3004

Supplementary information 1: Biopsy strategy

The table below summarises the biopsies taken from subjects according to intra-endoscopic diagnosis, and the subsequent application of this material. The strategy is also summarised in detail in our study design paper[1]

Diagnosis	Biopsy Position(s)	Other Samples
Normal	2x mid-sigmoid	Stool (for SCFA)<br>Biopsy for IHC
Adenoma	2x mid-sigmoid	Stool (for SCFA) Biopsy for IHC
	2x contralateral wall	Biopsy for IHC
	2x adenoma	Biopsy for IHC

[1] Corfe, B. M., Williams, E. A., Bury, J. P., Riley, S. A., Croucher, L. J., Lai, D. Y., . . . Evans, C. A. (2009). A study protocol to investigate the relationship between dietary fibre intake and fermentation, colon cell turnover, global protein acetylation and early carcinogenesis: the FACT study.. *BMC Cancer*, *9*, 332.

Supplementary information 2: Detailed iTRAQ workflow.

Lysis, pooling and fractionation

Colorectal pinch biopsies (~5mg) were suspended in 30µl of kinase buffer/mg in a Bertin CK14 homogenisation tube followed by homogenisation. The lysate was removed and centrifuged to separate the insoluble fraction and the supernatant (insoluble fraction). Biopsies were grouped by diagnosis and region and were ranked by faecal butyrate level and then pooled: eight biopsy lysates (two each from four patients) we used in each pool. Acetyl proteins were immunoprecipitated using antibody Ab 3879 (Chemicon Anti-acetyl lysine, rabbit polyclonal) antibody immobilised to a Pierce Seize column following manufacturer's directions. Samples were diluted in binding buffer, mixed over the antibody-gel slurry overnight and then unbound fraction was eluted by gravity flow. Bound fractions enriched for acetyl proteins were analysed by 2D Gel Electrophoresis (see below), the unbound soluble proteins were analysed by an iTRAQ workflow (see below). The insoluble fraction is principally intermediate filaments, and was extracted and solubilised by our adapted method[25] before analysis by iTRAQ. The workflow is summarised in Figure 1.

Peptide labeling with iTRAQ Reagents

Samples (100 µg each) were proteolytically digested with trypsin, reduced and cysteine blocked, then labeled according to procedures outlined by Applied Biosystems.

Peptide fractionation

Strong cation exchange was achieved using a PolySULFOETHYL A Pre-Packed Column (PolyLC, Columbia, MD) with a 5 µm particle size and a column dimension of 100 mm×4.6 mm i.d., 200 Å pore size, on a BioLC HPLC (Dionex, Surrey, U.K.). SCX was achieved using a low ionic buffer A (20% acetonitrile, 0.1% formic acid), a high ionic buffer B (20% acetonitrile, 0.1% formic acid, 500 mM KCl). Sample was loaded onto the column and washed for at least 60 minutes at a flow rate of 0.4 mL/min with 100 % SCX Buffer A (20 % acetonitrile, 0.1 % Formic Acid) to remove salts, TCEP and unincorporated iTRAQ reagent. Peptides were then separated using a gradient of SCX Buffer B (20% acetonitrile, 0.1% formic acid, 0.5 M KCl) at the same flow rate of 400 µL /min. Buffer B levels increased from 0% to 25% from 5 minutes to 30 minutes then from 25% to 100% over 5 minutes, followed by an increase from 26% to 100% over the next 15 min. Buffer B was held for another 5 min for isocratic washing prior to column re-equilibration with buffer A. The sample injection volume was 100 µL, and the liquid flow rate was 0.4 mL/min. The SCX chromatogram was monitored using UVD170U ultraviolet detector and Chromeleon software v. 6.50 (Dionex, LC Packings, The Netherlands). Fractions were collected using a Foxy Jr. (Dionex) fraction collector in 1 min intervals. Fractions were vacuum-concentration prior to LC-MS/MS analysis.

LC-MS/MS analysis

Fractions collected from offline separation techniques were eluted through the Famos-Ultimate 3000 nano-LC system (Dionex, LC Packings, The Netherlands) interfaced with a QSTAR XL (Applied Biosystems; MDS-Sciex) tandem ESI-QUAD-TOF MS. Vacuum dried fractions were resuspended in loading buffer (3% acetonitrile, 0.1% trifluoroacetic acid), injected and captured into a 0.3×5 mm trap column (3 μm C18 Dionex-LC Packings). Trapped samples were then eluted onto a 0.075×150 mm analytical column (3 μm C18 Dionex-LC Packings) using an automated binary gradient with a flow of 300 nL/min from 95% buffer A (3% acetonitrile, 0.1% formic acid), to 35% buffer B (97% acetonitrile, 0.1% formic acid) over 90 min, followed by a 5 min ramp to 95% buffer II (with isocratic washing for 10 min). Predefined 1 s 350−1600 m/z MS survey scans were acquired with up to two dynamically excluded precursors selected for a 3 s MS/MS (m/z 65−2000) scan. The collision energy range was increased by 20% as compared to the unlabeled peptides in order to overcome the stabilizing effect of the basic N-terminal derivatives, and to achieve equivalent fragmentation as recommended by Applied Biosystems.

Protein identification and relative quantification

The mass-spectrometric data was collected and analysed as previously described[2], [3], Briefly, MS/MS data generated from the QSTAR® XL was first converted to generic MGF peaklists using the mascot.dll embedded script (version 1.6 release no. 25) in Analyst QS v. 1.1 (Applied Biosystems, Sciex; Matrix Science). Further processing of the data was undertaken using an in-house Phenyx algorithm cluster (binary version 2.6; Geneva Bioinformatics SA) at the ChELSI Institute, University of Sheffield, against the *Homo sapiens* UniProt protein knowledgebase (SwissProt and Trembl (41070 and 71449 entries respectively, downloaded 5th November 2010,) to derive peptide sequence and hence protein identification. These data were then searched within the reversed *Homo sapiens* database to estimate the false-positive rate[4]. Peptides identifications at 1% false discovery rate were accepted. The iTRAQ reporter ion intensities were exported. Protein quantifications were obtained by computing the geometric means of the reporters' intensities. Median correction was subsequently applied to every reporter in order to compensate for systematic errors, *e.g.* if a sample happened to

---

[2] Pham, T.K., et al., A quantitative proteomic analysis of biofilm adaptation by the periodontal pathogen Tannerella forsythia. *Proteomics*, 2010. 10(17): p. 3130-41.

[3] Majumdar, D., Rosser, R., Havard, S., Lobo, A. J., Wright, P. C., Evans, C. A., Corfe, B. M. (2012, July). An integrated workflow for extraction and solubilization of intermediate filaments from colorectal biopsies for proteomic analysis.. *Electrophoresis, 33*(13), 1967-1974.

[4] Elias, J.E. and S.P. Gygi, Target-decoy search strategy for increased confidence in large-scale protein identifications by mass spectrometry. *Nat Methods*, 2007. 4(3): p. 207-14.

have been loaded at a largely different total concentration. The reporters' intensities, in each individual MS/MS scan, were also median corrected using the same factors, with the rationale that if the total concentration of a sample A was half that of another sample B, the intensities of sample A's reporter have to be doubled to allow for a fair comparison. *t*-tests applied to determine alterations in protein level between samples use these corrected intensities since these were was carried out for every protein and because of the multiple times each test was performed, the threshold (α=5%) used for significance was corrected for data mining. Here, we used the standard Bonferroni correction (α/*P*, where *P* is the number of proteins) to minimise false positive results. This workflow was developed in house[2].

Calculation of pI and MW

The physicochemical properties of peptides identified and relatively quantified in the analysis were plotted using the Innovagen peptide property calculator.

Supplementary information 3: Detailed 2d gel method

Protein lysates were prepared from frozen patient mononuclear cell material, which was resuspended in 350 µl of isoelectric focusing buffer (9 m urea, 2 m thiourea, 4% (w/v) CHAPS, 65 mm dithiothreitol, 0.5% (v/v) IPG buffer (Amersham Biosciences)) per 1 × 106 cells. Isoelectric focusing was performed using 18-cm immobilized pH gradient strips (pH 3-10 NL) and the Multiphor II instrument (Amersham Biosciences), focusing for a total of 49 kV h at 20 °C. The second dimension was a standard SDS-PAGE protocol using the ISODALT system (Amersham Biosciences). Strips were equilibrated for 10 min in equilibration buffer (50 mm Tris-HCl, pH 6.8, 6 m urea, 30% (v/v) glycerol, 2% (v/v) SDS) containing 65 mm dithiothreitol and then for 10 min in the same buffer containing 240 mm iodoacetamide. Second-dimension gels were 12% SDS-PAGE gels of 160 × 180 × 0.75 mm. Gels were stained with silver using the protocol of Shevchenko et al[5].

[5] Shevchenko, A., Wilm, M., Vorm, O., and Mann, M. (1996) Mass spectrometric sequencing of proteins silver-stained polyacrylamide gels. Anal. Chem. 68, 850– 858

Supplementary information 4: Summary of Patient data

Subject Characteristics			
	**Normal**	**Adenoma**	***p***
**n**	**34**	**28**	
**Age (yr)**	**62.5±11.4**	**68.1±10.1**	**0.047**
**Weight (Kg)**	**82.6± XX**	**78.2±11.6**	**0.247**
**BMI (Kg/m2)**	**26.4±4.8**	**25.5 ±3.4**	**0.404**

Adenoma Characteristics				
**<10mm /≥10mm**	**13/14**			
**Synchronous adenoma**	**3**			
**Caecal**	**Right sided**	**Sigmoid**	**Recosigmoid junction**	**Rectal**
**3**	**3**	**5**	**2**	**2**

Supplementary Information 5: Table of proteins identified in the IF Dataset

Ascension number	Number of peptides matched	Protein name
A2NUT2_CHAIN_0	1	Lambda chain
A4D1Z4	1	KIA00415 gene product
A6NN01	1	histone A2A
B0YJC4	1	vimentin
B3KSN3	1	C DNA (highly similar to ATP-binding cassette sub-family B member 8, mitochondrial)
B4DGF3	1	C DNA (highly similar to Talin-2)
B4DIK9	1	C DNA
B4DRV1	1	C DNA (highly similar to Protein-glutamine gamma-glutamyltransferase K)
B4DRX3	1	60S ribosomal protein
B4DU60	1	Citrate lyase subunit beta-like protein, mitochondrial
B4DUI9	1	C DNA (highly similar to Troponin C, skeletal muscle)
B5MEB8	1	obsolete
B7Z1I0	1	integrin linked protein kinase
B7Z2X4	1	C DNA (highly similar to Gelsolin)
B8ZZ37	1	obsolete
D2CFK5	1	somatostatin receptor 5C
D3YTB1	1	60S ribosomal protein
P05141_CHAIN_0	1	ADP/ATP translocase 2
P06733_CHAIN_0	1	Alphaenolase
P26599	1	Polypyrimidine tract binding protein
P35268_CHAIN_0	1	60S ribosomal protein

P50914_CHAIN_0	1	60S ribosomal protein
P51884_CHAIN_0	1	Lumican
P62269_CHAIN_0	1	40s ribosomal protein
P98160_CHAIN_0	1	basement membrane
Q15746_ISOFORM_3B	1	myosin light chain
Q3B7J3	1	ZCCHC3 protein
Q53S60	1	putatative uncharacterised protein
Q6IBG5	1	MYL6 protein
Q6NUK4_ISOFORM_2	1	Receptor expression-enhancing protein 3
Q71S07	1	Non-erythrocytic beta-spectrin 4
Q765P7_ISOFORM_2	1	Actin-bundling with BAIAP2 homology protein 1
Q8N7L7	1	C DNA (FLJ40893 fis, clone UTERU200160)
Q8WXQ3	1	putatative uncharacterised protein
Q96S66_ISOFORM_4	1	Chloride channel CLIC-like protein 1
Q9BYE0	1	Transcrition factor HES-7
Q9H0N0	1	Ras-related protein Rab-6C
Q9H6H4_ISOFORM_2	1	Receptor expression-enhancing protein 4
Q9NYP9	1	RER1 Protein
Q9UED0	1	amyloid like protein 2
A8K230	2	zinc finger protein
B4DJ98	2	C DNA (highly similar to Protein disulfide-isomerase A3)
B4DJC3	2	Histone H2A
B4DPR2	2	C DNA (highly similar to Serum albumin)
B4DRD6	2	Histone H1
B7Z3F2	2	C DNA

B7Z3U6	2	sodium pump subunit alpha 1
C9JA88	2	obsolete
C9JRX8	2	LYR motif containing protein 4
D3DP13	2	fibrinogen beta chain
P07355_CHAIN_0	2	Annexin A2
P07585_CHAIN_0	2	Decorin
P08727	2	K19
P11021_CHAIN_0	2	78 kDA glucose related protein
P15924	2	Desmoplakin
P54707	2	potassium transporting ATPase alpha 2
Q01082_ISOFORM_2	2	Spectrin beta chain
Q3MIV8	2	myosin heavy chain 11
Q59GW6	2	Acetyl-CoA acetyltransferase, cytosolic variant
Q6DD88	2	atlastin-3
Q9P0H9	2	Ribosome binding protein1
Q9P2E9_ISOFORM_1	2	Ribosome binding protein1
Q9Y4F5_ISOFORM_3	2	Protein KIAA0284
Q9Y6C2_CHAIN_0	2	Elastin microfibril interface-located protein 1
A6NKY3	3	obsolete
A8K092	3	ATP synthase subunit alpha
B2R4U6	3	C DNA
P02545_ISOFORM_ADelta10	3	Prelamin
P08572	3	collagen alpha 2 chain
P16401_CHAIN_0	3	Histone H1.5
P46782_CHAIN_1	3	40s ribosomal protein
P62851	3	40s ribosomal protein
B4E335	4	Actin

P62277_CHAIN_0	4	40s ribosomal protein
Q12959_ISOFORM_5	4	disks large homolg
P01857	5	Ig gamma chain region 1C
P05783_CHAIN_0	5	K18
P12111_ISOFORM_2	5	collagen alpha 3 chain
P35579_ISOFORM_2	5	Myosin 9
B7Z9B0	7	C DNA growth arrest specific protein 8
P02461_CHAIN_0	8	collagen alpha 1 chain
Q53SW3	8	putatative uncharacterised protein
Q5HY54	8	filamin A
P05787_CHAIN_0	10	K8
Q702N8_ISOFORM_B	10	Xin actin-binding repeat-containing protein 1
Q9HAM5	10	C DNA (moderately similar to HYPOXIA-INDUCIBLE FACTOR 1 ALPHA)
Q9NRC6	10	Spectrin beta chain
P12109_CHAIN_0	11	collagen alpha 1 chain
Q14222	12	EEF1A
P58876_CHAIN_0	14	Histone H2B
P68431_CHAIN_0	18	histone H3.1
P50591	27	TNF superfamily lignd 10
P02452_CHAIN_0	37	collagen alpha 1 chain
P08123_CHAIN_0	60	collagen alpha 2 chain
P62805_CHAIN_0	119	histone

Supplementary Information 6: Protein interaction network for the IF Dataset

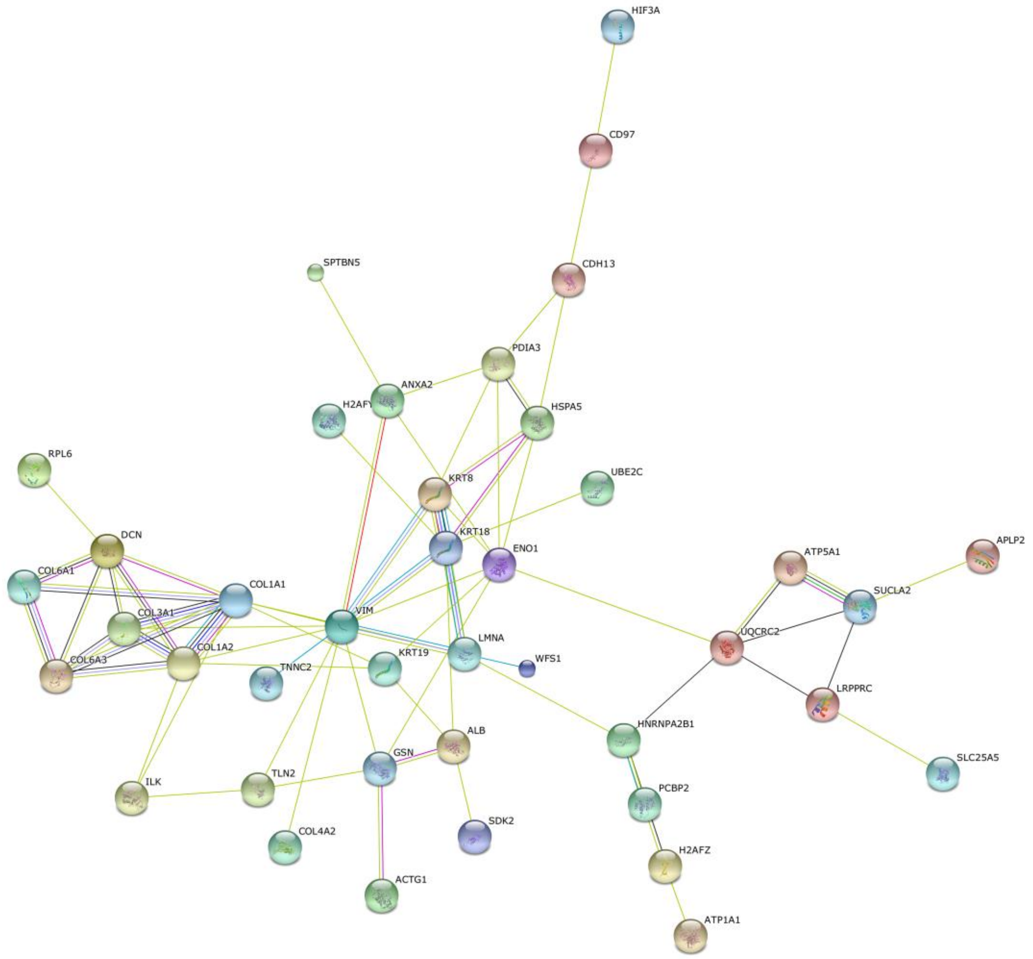

Protein identifiers for the IF fraction were submitted to STRINGS database. Orphan nodes were excluded from the representation. All remaining proteins formed a potential single network, centred on the clusters around keratin 8, 18 and vimentin and the collagens. Whilst caution is needed as some edges are based solely on text mining, the data suggest a surprising cohesiveness.

Supplementary Information 7: Analysis of pathways representation for IF Dataset

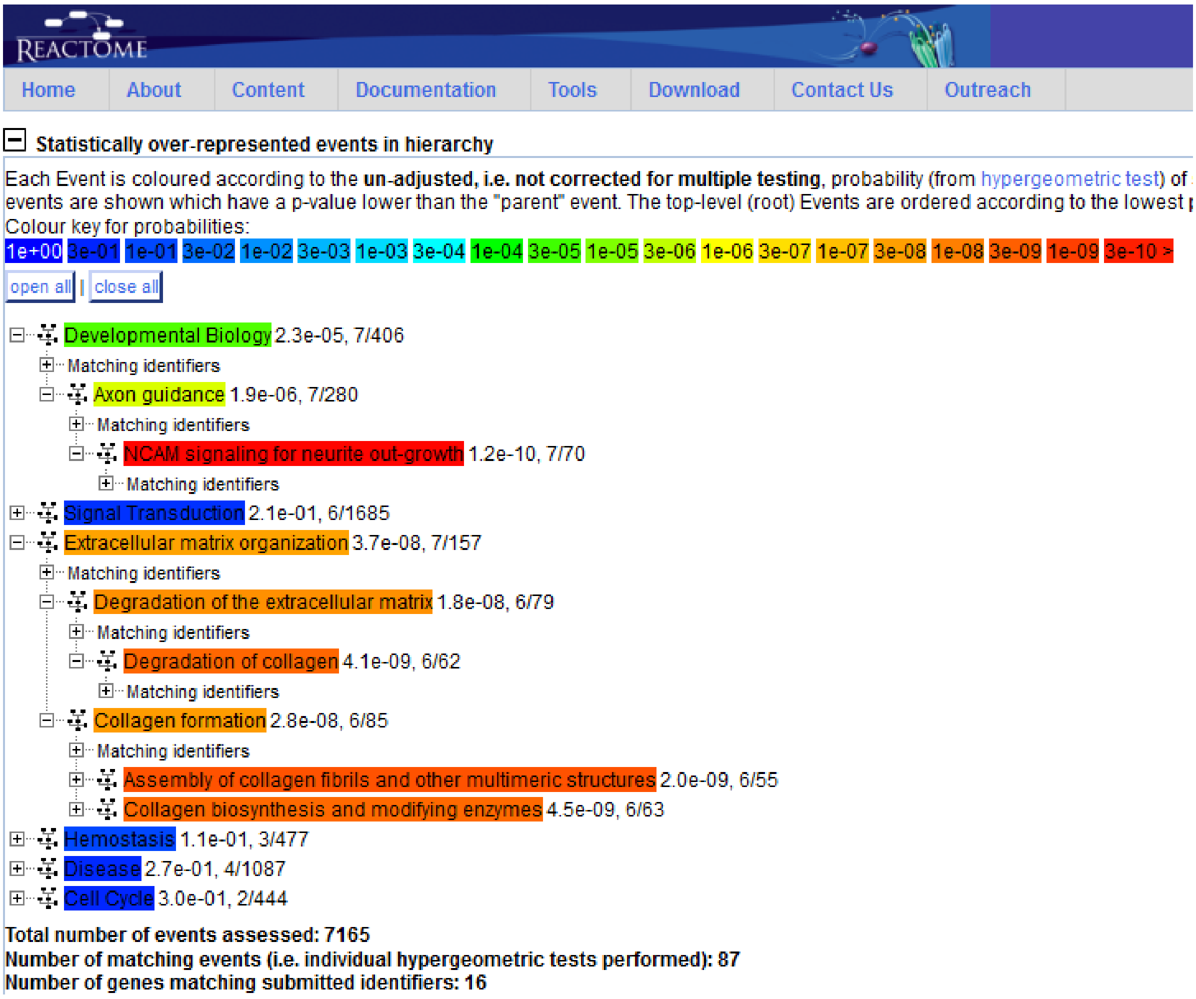

The entire sampleset listed in the table in section 5 was assessed using the pathways analysis tool in Reactome (www.reactome.org) on 18th February 2012. The Pathways identified as enriched are colour-coded as a heat-map and assigned a P-value following hypergeometric analysis. The data suggest that NCAM signalling for neurite outgrowth is highly over-represented (P>1.2 x10^{-10}). Other pathways over represented include collagen formation and modification. As the dataset are skewed for cytoskeletal and insoluble proteins, this is confirmatory that the fractionation is enriching correctly.

A tabulated version of these data is shown below.

Un-adjusted probability of seeing N or more genes in this Event by chance	Number of proteins in sample which map to this Event	Total number of proteins involved in this Event	Name of this Event
2.73E-12	6	20	Interaction of NCAM1 with collagens
1.61E-11	6	26	PDGF binds to extracellular matrix proteins
1.22E-10	7	70	NCAM signaling for neurite out-growth
3.60E-10	6	42	Secretion of collagens
4.83E-10	6	44	NCAM1 interactions
4.83E-10	6	44	Association of procollagen chains
5.56E-10	6	45	PDI is a chaperone for collagen peptides
6.39E-10	6	46	Galactosylation of collagen propeptide hydroxylysines by PLOD3
6.39E-10	6	46	Glucosylation of collagen propeptide hydroxylysines
7.31E-10	6	47	Galactosylation of collagen propeptide hydroxylysines by procollagen galactosyltransferases 1, 2.
8.35E-10	6	48	Collagen prolyl 4-hydroxylase converts proline to 4-hydroxyproline
8.35E-10	6	48	Procollagen lysyl hydrolases convert lysine to 5-hydroxylysine
8.35E-10	6	48	Procollagen triple helix formation
1.08E-09	6	50	Collagen prolyl 3-hydroxylase converts proline to 3-hydroxyproline
1.95E-09	6	55	Assembly of collagen fibrils and other multimeric structures
4.10E-09	6	62	Degradation of collagen
4.53E-09	6	63	Collagen biosynthesis and modifying enzymes
1.81E-08	6	79	Degradation of the extracellular matrix
2.83E-08	6	85	Collagen formation
3.72E-08	7	157	Extracellular matrix organization
5.48E-08	4	17	Gelatin degradation by MMP19
2.42E-07	4	24	Gelatin degradation by MMP1, 2, 3, 7, 8, 9, 12, 13
1.93E-06	3	11	Formation of collagen fibrils
1.93E-06	3	11	Formation of collagen fibres
1.95E-06	7	280	Axon guidance
2.57E-06	3	12	Collagen type VII binds laminin-322 and collagen IV
2.91E-06	6	185	Signaling by PDGF

4.24E-06	3	14	Formation of collagen networks
5.30E-06	3	15	Removal of fibrillar collagen N-propeptides
5.30E-06	3	15	Removal of fibrillar collagen C-propeptides
5.30E-06	3	15	Anchoring fibril formation
5.56E-06	2	2	Formation of histidino-hydroxylysinonorleucine cross-links
5.56E-06	2	2	Formation of dehydro-lysinonorleucine cross-links
5.56E-06	2	2	Formation of lysyl-pyrrole cross-links
5.56E-06	2	2	Formation of hydroxylysyl-pyridinoline cross-links
5.56E-06	2	2	Formation of dehydro-hydroxylysino-norleucine cross-links
5.56E-06	2	2	Formation of hydroxylysino-5-ketonorleucine cross-links
5.56E-06	2	2	Formation of lysino-5-ketonorleucine cross-links
5.56E-06	2	2	Formation of lysyl-pyridinoline cross-links
5.56E-06	2	2	Formation of hydroxylysyl-pyrrole cross-links

Supplementary Information 8: Soluble proteins entering iTRAQ workflow

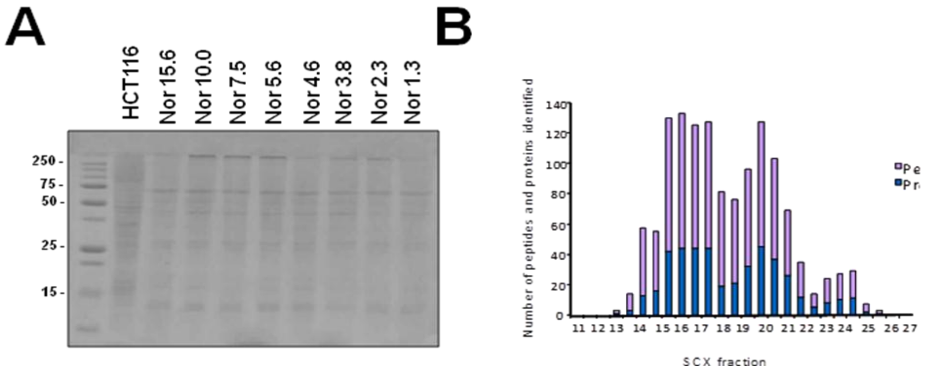

Characterisation of proteins in IP unbound fraction

The abundance and variation in proteins present in the unbound IP fraction was determined by SDS-PAGE and silver-staining (Panel A). The figure shows large numbers of protein species in the sample, with a representative cross-section of molecular weights. These data demonstrate that the sample remains in good condition (little degradation) and is not skewed to species of any particular mass range. Samples were tryptic digested, iTRAQ-labelled and separated by SCX. The distribution of peptides/proteins across the SCX fractionation is shown in Panel B. In total 183 proteins were identified using this approach.

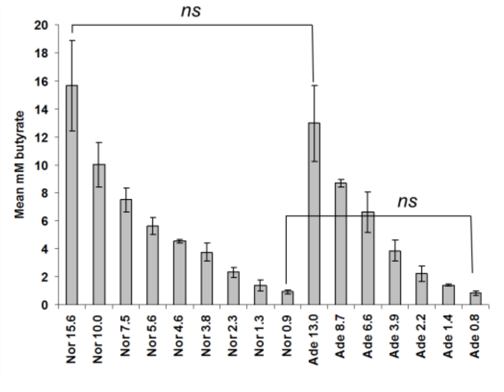

Panel C shows the pooling strategy. Subjects were stratified by diagnosis (Nor-normal, Ade-adenoma) and by the concentration of butyrate in faecal samples. Four subjects' worth of biopsies were required for a successful IP (data not shown). Mean butyrate level of subjects' faecal SCFA was determined for each pool, pools are signified by the diagnosis and mean butyrate, shown on the x-axis. There was no significant difference in the mean butyrate levels stools of the highest and lowest pools (T-test).

Supplementary information 9: Table of proteins identified using the iTRAQ workflow on soluble protein extract

Accession	*Score*	*#valid pept sequences*	*% Cov*	*Mass Da*	*pI*	*# peptides quant*	*Description*
P31946_ISOFORM_Short	42.5	6	18	27850	4.8	8	14-3-3 protein beta/alpha, N-terminally processed [ISOFORM Sho
P25398_CHAIN_0	7.4	1	8	14384	7.7	1	40S ribosomal protein S12 [CHAIN 0]
P10809_CHAIN_0	25.9	4	8	57963	5.3	1	60 kDa heat shock protein, mitochondrial (HSP-60) (Hsp60) (CPN
P11021_CHAIN_0	18.3	3	5	70479	5.0	4	78 kDa glucose-regulated protein (GRP-78) (BiP) [CHAIN 0]
P00326_CHAIN_0	17.8	3	7	39736	8.7	5	Alcohol dehydrogenase 1C [CHAIN 0]
Q13747	22.5	3	15	22828	6.3	4	Alpha-1 antitrypsin;
P02763_CHAIN_0	22.7	2	13	21560	5.1	8	Alpha-1-acid glycoprotein 1 (AGP 1) (OMD 1) [CHAIN 0]
P19652_CHAIN_0	18.1	2	10	21651	5.2	2	Alpha-1-acid glycoprotein 2 (AGP 2) (OMD 2) [CHAIN 0]
P02765_CHAIN_0	8.4	1	4	30222	4.6	1	Alpha-2-HS-glycoprotein chain B [CHAIN 0]
O43707	51.0	7	8	104854	5.3	5	Alpha-actinin-4
P06733_CHAIN_0	60.6	8	16	47038	7.6	18	Alpha-enolase (NNE) [CHAIN 0]
P07355_CHAIN_0	14.1	2	6	38473	8.1	2	Annexin A2 (PAP-IV) [CHAIN 0]
P08758_CHAIN_0	23.3	3	11	35806	5.0	1	Annexin A5 (CBP-I) (PAP-I) (PP4) (VAC-alpha) [CHAIN 0]
Q5EFE6_CHAIN_0	29.4	3	17	23495	8.6	5	Anti-RhD monoclonal T125 kappa light chain; [CHAIN 0]
P02647_CHAIN_1	51.7	7	28	27950	5.4	17	Apolipoprotein A-I(1-242) [CHAIN 1]
A8K092	62.0	9	18	54494	8.5	13	ATP synthase subunit alpha
Q0QEN7	40.1	5	16	48113	5.0	6	ATP synthase subunit beta
B7Z3U6	21.4	3	3	109550	5.2	4	ATPase, Na+/K+ transporting, alpha 1 polypeptide, isoform CRA_
P61769_CHAIN_1	13.7	2	16	11618	6.5	3	Beta-2-microglobulin form pI 5.3 [CHAIN 1]
Q71UM7	9.8	1	8	13869	4.8	4	Beta-subunit signal transducing proteins GS/GI;
Q12864_CHAIN_0	30.2	4	5	89761	5.0	4	Cadherin-17 (LI-cadherin) [CHAIN 0]
A8K7I4_CHAIN_0	68.1	8	9	97914	6.0	14	Calcium-activated chloride channel regulator 1 (hCLCA1) (CaCC-1
Q9BRL5	13.8	2	12	16507	4.4	3	CALM3 protein;
P27797_CHAIN_0	35.4	6	12	46466	4.3	1	Calreticulin (ERp60) [CHAIN 0]
P00915_CHAIN_0	20.2	3	12	28739	6.9	8	Carbonic anhydrase 1 (CA-I) (CAB) [CHAIN 0]
P00918_CHAIN_0	17.7	3	13	29115	7.2	4	Carbonic anhydrase 2 (CA-II) (CAC) [CHAIN 0]
O00748_CHAIN_0	6.6	1	2	58951	5.4	1	Carboxylesterase 2 (CE-2) (hCE-2) [CHAIN 0]
P07339_CHAIN_0	15.1	2	6	37852	5.8	5	Cathepsin D heavy chain [CHAIN 0]
Q9H5A3	14.9	2	8	29391	5.1	1	CD44 molecule (Indian blood group);
B3KML9	70.1	8	19	44602	4.9	22	cDNA FLJ11352 fis, clone HEMBA1000020, highly similar to Tubul
Q9NXQ7	13.5	2	7	31778	9.5	4	cDNA FLJ20106 fis, clone COL04830;
Q9NXM7	7.8	1	5	16519	8.3	2	CDNA FLJ20154 fis, clone COL08740;
B3KPS3	41.0	4	13	46241	5.0	13	cDNA FLJ32131 fis, clone PEBLM2000267, highly similar to Tubuli
B3KSG9	10.1	1	2	65431	7.2	1	cDNA FLJ36188 fis, clone TESTI2027179, highly similar to Transm
B3KSV9	15.6	2	7	29437	6.5	2	cDNA FLJ37148 fis, clone BRACE2025333, highly similar to Homo
B3KT41	6.2	1	1	77279	4.9	1	cDNA FLJ37598 fis, clone BRCOC2008642, highly similar to Synap
B3KTT5	23.2	3	7	51917	5.4	4	cDNA FLJ38698 fis, clone KIDNE2002015, highly similar to HEAT S
B4DW73	19.5	3	5	55953	6.9	3	cDNA FLJ50710, highly similar to Phosphoenolpyruvate carboxyk
B4E1S2	12.0	1	8	15645	5.0	1	cDNA FLJ51185, highly similar to Annexin A4;
B4DRT4	6.5	1	5	17326	5.8	2	cDNA FLJ51535, highly similar to Phosphatidylethanolamine-bin

B4DP56	31.2	4	12	38694	5.3	8	cDNA FLJ52237, highly similar to Creatine kinase B-type (EC 2.7.3
B4DL87	16.2	2	10	18536	6.9	1	cDNA FLJ52243, highly similar to Heat-shock protein beta-1;
B4DNR3	8.8	1	5	19797	6.1	1	cDNA FLJ52710, highly similar to Abhydrolase domain-containing
B4E335	108.6	12	36	39226	5.5	44	cDNA FLJ52842, highly similar to Actin, cytoplasmic 1;
B7Z504	6.6	1	5	18406	10.1	1	cDNA FLJ52950;
B4DFY0	29.5	4	8	61575	5.1	1	cDNA FLJ53313, highly similar to Alpha-actinin-1;
B7Z2X4	26.2	4	5	77789	5.5	5	cDNA FLJ53327, highly similar to Gelsolin;
B4DP93	21.2	3	4	87544	5.5	3	cDNA FLJ53437, highly similar to Major vault protein;
B4E2L8	7.6	1	2	70344	5.2	1	cDNA FLJ53487, highly similar to Coagulation factor XIII A chain (I
B7Z6P1	94.7	11	33	38579	5.3	1	cDNA FLJ53662, highly similar to Actin, alpha skeletal muscle;
B4E1B2	75.3	10	17	74832	7.0	12	cDNA FLJ53691, highly similar to Serotransferrin;
B4DQG5	7.7	1	2	39346	9.5	1	cDNA FLJ54122, highly similar to Cytosol aminopeptidase (EC 3.4
B4DZ95	7.2	1	2	92772	6.5	1	cDNA FLJ54570, highly similar to 2-oxoglutarate dehydrogenase I
B4DLK2	7.1	1	9	13098	5.9	1	cDNA FLJ55097, highly similar to Adenylate kinase isoenzyme 2, r
B4DSS4	8.3	1	1	106116	5.7	4	cDNA FLJ56631;
B4DKN9	13.2	2	9	19570	8.7	1	cDNA FLJ57740, highly similar to Transforming protein RhoA;
B4DDF3	6.4	1	4	30029	9.7	1	cDNA FLJ58050, highly similar to Interleukin enhancer-binding fa
B4E1R7	11.5	2	3	60507	5.0	1	cDNA FLJ58224, highly similar to Calpain-2 catalytic subunit (EC 3
B4DQ92	8.6	1	2	49616	4.7	1	cDNA FLJ59379, highly similar to Hematopoietic lineage cell-spec
B4DKG3	6.2	1	5	12169	8.6	1	cDNA FLJ60631, moderately similar to Scaffold attachment factor
B4DTV8	6.6	1	1	135871	5.1	1	cDNA FLJ61399, highly similar to Spectrin alpha chain, brain;
B3KQT9	36.5	5	10	54102	7.1	6	cDNA PSEC0175 fis, clone OVARC1000169, highly similar to Prote
B2R4P2	11.9	2	10	22201	8.8	2	cDNA, FLJ92164, highly similar to Homo sapiens peroxiredoxin 1 (
B2R4V4	7.1	1	14	10016	6.6	2	cDNA, FLJ92232, highly similar to Homo sapiens barrier to autoin
B2R5M8	27.3	3	7	46649	6.8	4	cDNA, FLJ92536, highly similar to Homo sapiens isocitrate dehydr
B2R9J0	6.8	1	2	53500	6.2	1	cDNA, FLJ94418, highly similar to Homo sapiens inositol 1,4,5-tris
B2RB23	12.9	2	5	42009	8.7	1	cDNA, FLJ95265, highly similar to Homo sapiens acetyl-Coenzyme
Q9UNM1	28.7	4	31	10295	9.3	21	Chaperonin 10-related protein;
Q0QEL2	13.2	2	10	24926	6.6	4	Citrate synthase
Q549M8	7.0	1	3	28068	6.4	1	CLE7;
P23528_CHAIN_0	34.0	5	29	18371	8.5	5	Cofilin-1 (p18) [CHAIN 0]
P12532_CHAIN_0	22.1	4	11	43080	7.8	1	Creatine kinase U-type, mitochondrial (U-MtCK) (Mia-CK) [CHAIN
C6KXN3	14.2	2	7	24742	5.7	1	Cyclosporin A transporter 1;
P04080	20.6	3	34	11140	7.9	8	Cystatin-B
P13073_CHAIN_0	13.3	2	14	17200	9.3	4	Cytochrome c oxidase subunit 4 isoform 1, mitochondrial (COX IV
P20674_CHAIN_0	8.0	1	9	12501	5.0	1	Cytochrome c oxidase subunit 5A, mitochondrial [CHAIN 0]
P53634_CHAIN_1	11.1	1	7	18473	6.2	1	Dipeptidyl peptidase 1 light chain [CHAIN 1]
P68104	45.7	6	15	50141	9.2	9	Elongation factor 1-alpha 1 (EF-1-alpha-1) (eEF1A-1) (EF-Tu)
P30040_CHAIN_0	7.2	1	4	25853	6.9	1	Endoplasmic reticulum resident protein 29 (ERp29) (ERp31) (ERp
P14625_CHAIN_0	87.3	12	13	90178	4.7	10	Endoplasmin (GRP-94) [CHAIN 0]
P10645_PEPT_12	8.8	1	24	4233	4.8	2	ER-37 [PEPTIDE 12])
Q5HY54	29.7	5	2	276550	5.7	1	Filamin A, alpha (Actin binding protein 280);
O75369_ISOFORM_5	20.2	3	1	230320	5.4	1	Filamin-B (FLN-B) (Truncated ABP) (Fh1) [ISOFORM 5]
P04075_CHAIN_0	47.7	6	16	39289	8.5	10	Fructose-bisphosphate aldolase A [CHAIN 0]
Q86TY5	38.4	5	41	13897	9.5	15	Full-length cDNA clone CS0DI041YE05 of Placenta of Homo sapie
P09382_CHAIN_0	14.2	2	15	14585	5.6	2	Galectin-1 (Gal-1) (HLBP14) [CHAIN 0]

P56470	46.6	6	16	35941	9.3	18	Galectin-4 (Gal-4) (L36LBP)
Q5TGM9	6.9	1	10	13291	5.8	2	GDP-mannose 4,6-dehydratase;
B4DE36	10.0	1	3	60186	8.4	1	Glucose-6-phosphate isomerase
Q14400	16.5	2	11	28695	8.4	2	GLUD1 protein;
Q5TA02	6.7	1	5	23341	7.6	1	Glutathione S-transferase omega 1;
P04406_CHAIN_0	61.2	8	24	35922	8.8	13	Glyceraldehyde-3-phosphate dehydrogenase (GAPDH) [CHAIN 0]
P11216_CHAIN_0	28.2	4	6	96565	6.5	4	Glycogen phosphorylase, brain form [CHAIN 0]
P00738_CHAIN_2	29.1	5	17	27265	6.5	5	Haptoglobin beta chain [CHAIN 2]
B4DMJ7	8.5	1	9	14526	5.9	1	HCG2015269, isoform CRA_c;
P07900_CHAIN_0	44.0	6	7	84529	5.0	6	Heat shock protein HSP 90-alpha (HSP 86) (HSP86) [CHAIN 0]
P08238_CHAIN_0	45.4	6	8	83133	5.0	13	Heat shock protein HSP 90-beta (HSP 90) (HSP 84) (HSP84) [CHAI
P69905_CHAIN_0	40.2	4	31	15126	9.1	30	Hemoglobin subunit alpha [CHAIN 0]
Q8IZP7	6.5	1	2	54844	6.5	2	Heparan-sulfate 6-O-sulfotransferase 3 (HS6ST-3)
Q9NYD7	9.3	1	24	5865	9.9	1	High mobility group 1 protein;
O00479_CHAIN_0	6.6	1	8	9408	10.6	1	High mobility group nucleosome-binding domain-containing prot
Q96IS6	32.2	5	11	64673	5.4	5	HSPA8 protein;
P54868_CHAIN_0	32.8	4	9	52383	7.0	6	Hydroxymethylglutaryl-CoA synthase, mitochondrial (HMG-CoA s
P01876	56.0	6	17	37655	6.3	22	Ig alpha-1 chain C region
P01857	33.8	4	13	36106	8.6	5	Ig gamma-1 chain C region
P01860	37.5	5	15	41287	8.4	10	Ig gamma-3 chain C region
P01871	20.3	3	7	49307	6.6	2	Ig mu chain C region
Q9Y6R7_CHAIN_0	30.9	4	1	569345	5.2	9	IgGFc-binding protein (FcgammaBP) [CHAIN 0]
Q6P5S8	34.5	4	19	25773	6.3	11	IGK@ protein;
P48735	22.6	3	6	50909	9.0	1	Isocitrate dehydrogenase [NADP], mitochondrial (IDH)
P05783_CHAIN_0	30.0	5	10	47927	5.4	2	Keratin, type I cytoskeletal 18 (CK-18) (K18) [CHAIN 0]
P08727	79.7	8	20	44092	5.1	17	Keratin, type I cytoskeletal 19 (CK-19) (K19)
P05787_CHAIN_0	93.7	13	25	53573	5.6	25	Keratin, type II cytoskeletal 8 (CK-8) (K8) [CHAIN 0]
O60382	6.9	1	1	191307	11.9	1	KIAA0324;
Q5TCJ4	8.0	1	2	53250	6.2	2	Lamin A/C;
Q6NVH9	37.0	5	20	26197	8.9	1	Lectin, galactoside-binding, soluble, 3;
P68871_CHAIN_0	87.5	8	59	15867	7.3	80	LVV-hemorphin-7 [CHAIN 0]
Q0QF37	38.5	6	17	31969	8.4	7	Malate dehydrogenase
Q16853_CHAIN_0	13.5	2	2	84491	6.1	4	Membrane primary amine oxidase (SSAO) (VAP-1) [CHAIN 0]
Q02817_CHAIN_0	35.0	6	1	538420	5.5	2	Mucin-2 (MUC-2) [CHAIN 0]
Q6IBG5	8.4	1	8	12970	4.7	2	MYL6 protein;
P35579_CHAIN_0	41.0	6	4	226401	5.5	6	Myosin-9 (NMMHC II-a) (NMMHC-IIA) (NMMHC-A) [CHAIN 0]
O95178_CHAIN_0	6.3	1	14	8642	4.4	1	NADH dehydrogenase [ubiquinone] 1 beta subcomplex subunit 2
Q14697_CHAIN_0	31.5	4	5	103975	5.6	3	Neutral alpha-glucosidase AB [CHAIN 0]
P10153_CHAIN_0	10.9	1	11	15464	9.4	3	Non-secretory ribonuclease (RNase 2) [CHAIN 0]
P62937_CHAIN_0	45.9	7	38	17881	8.4	11	Peptidyl-prolyl cis-trans isomerase A (PPIase A) [CHAIN 0]
P30044_CHAIN_0	19.4	2	14	17031	7.7	11	Peroxiredoxin-5, mitochondrial (Prx-V) (AOEB166) [CHAIN 0]
B4E1H9	12.9	2	5	35046	8.7	4	Phosphoglycerate kinase
P68402	6.6	1	4	25569	5.7	1	Platelet-activating factor acetylhydrolase IB subunit beta (PAF-AH
Q9BXV5	7.6	1	7	22739	6.0	1	PNAS-139;
P07737_CHAIN_0	41.8	5	43	14923	8.8	18	Profilin-1 [CHAIN 0]
Q06323	19.0	3	12	28723	6.0	2	Proteasome activator complex subunit 1 (PA28alpha) (PA28a) (RE

B4E0X6	9.7	1	9	14624	9.1	1	Proteasome subunit alpha type
P07237_CHAIN_0	41.1	7	13	55294	4.7	5	Protein disulfide-isomerase (PDI) [CHAIN 0]
Q99497	6.6	1	4	19891	6.8	4	Protein DJ-1
Q5VWP2	6.5	1	2	44944	5.5	2	Protein FAM46C
Q2VXS9	7.0	1	1	76755	6.3	1	Proto-oncogene c-fes variant 2;
C9J3E2	17.8	2	14	15014	9.2	4	Putative uncharacterized protein AGR2;
C9JFR7	14.6	1	11	11333	9.8	4	Putative uncharacterized protein CYCS;
Q9NTC4	14.5	2	4	47557	4.8	1	Putative uncharacterized protein DKFZp434K0126;
Q9Y436	6.2	1	3	20483	5.7	1	Putative uncharacterized protein DKFZp586M2023;
Q5JPE4	6.4	1	6	20393	6.5	1	Putative uncharacterized protein DKFZp667O202;
Q68DG4	16.3	2	8	21507	7.9	6	Putative uncharacterized protein DKFZp686A15170;
C9J7B5	6.7	1	11	11945	8.6	2	Putative uncharacterized protein EIF5A2;
A8MW49	27.6	4	28	13808	5.5	5	Putative uncharacterized protein FABP1;
A8MX94	12.2	2	9	19480	6.1	1	Putative uncharacterized protein GSTP1;
C9JEY0	14.5	2	12	16129	10.2	3	Putative uncharacterized protein HADHB;
B8ZZ37	23.2	3	13	34202	9.1	4	Putative uncharacterized protein HNRNPA2B1;
C9JA05	6.3	1	11	8168	9.2	1	Putative uncharacterized protein IGJ;
D3YTI4	19.4	3	12	26712	8.6	3	Putative uncharacterized protein LDHA;
C9J9K3	16.9	2	8	29506	5.3	3	Putative uncharacterized protein RPSA;
A6NMW4	6.8	1	7	18342	7.8	1	Putative uncharacterized protein SNX12;
C9J0K6	15.8	2	12	17605	5.8	4	Putative uncharacterized protein SRI;
C9JGI3	7.2	1	3	46087	5.5	1	Putative uncharacterized protein TYMP;
C9JTQ8	8.0	1	1	58852	10.1	1	Putative uncharacterized protein ZNF584;
Q8WUW7	28.4	4	14	37276	8.7	6	Pyruvate kinase
Q8WVC2	8.4	1	11	8850	9.1	1	RPS21 protein;
D3DV39	24.6	4	34	10180	5.6	1	S100 calcium binding protein A6 (Calcyclin), isoform CRA_a;
Q13228_ISOFORM_2	41.8	5	15	45349	6.1	6	Selenium-binding protein 1 (SBP56) (SP56) [ISOFORM 2]
P02768_CHAIN_0	221.3	28	38	66472	5.7	121	Serum albumin [CHAIN 0]
Q9Y566_ISOFORM_2	6.1	1	1	158792	7.1	8	SH3 and multiple ankyrin repeat domains protein 1 (Shank1) (SST
Q59EP7	16.3	2	8	29347	9.8	5	Solute carrier family 25 member 4 variant;
B4E3K9	9.2	1	9	18262	8.8	2	Superoxide dismutase
A8MST3	6.8	1	5	13909	5.9	2	Superoxide dismutase [Cu-Zn]
P23381_CHAIN_2	9.4	1	4	43329	7.6	2	T2-TrpRS [CHAIN 2]
O60744	22.8	3	37	9321	6.7	9	Thioredoxin delta 3;
P30048_CHAIN_0	7.3	1	6	21468	6.0	2	Thioredoxin-dependent peroxide reductase, mitochondrial (Prx-I
A8MW06_PEPT_0	15.1	2	30	4931	5.3	1	Thymosin beta-4-like protein 3 [PEPTIDE 0])
Q5VU62	39.4	6	30	18493	4.7	2	TPM3 protein;
Q53GC9	27.8	4	21	20889	9.0	6	Transgelin variant;
P37802_CHAIN_0	45.3	6	23	22260	8.8	4	Transgelin-2 [CHAIN 0]
Q53WY6	10.3	1	30	4570	9.2	1	Transthyretin;
Q07654_CHAIN_0	7.7	1	19	6580	5.7	2	Trefoil factor 3 (hITF) (hP1.B) [CHAIN 0]
P60174_CHAIN_0	36.1	4	19	26538	7.1	13	Triosephosphate isomerase (TIM) [CHAIN 0]
O14773_CHAIN_0	15.4	2	7	39790	5.9	1	Tripeptidyl-peptidase 1 (TPP-1) (TPP-I) (LPIC) [CHAIN 0]
P09493_ISOFORM_6	42.8	7	19	32649	4.7	2	Tropomyosin alpha-1 chain [ISOFORM 6]
P67936_ISOFORM_2	46.4	7	20	32723	4.7	5	Tropomyosin alpha-4 chain [ISOFORM 2]
D2E6S1	6.6	1	5	25031	6.4	2	Tryptase beta I;

P62988	16.3	2	20	8565	8.1	5	Ubiquitin
Q16851_ISOFORM_2	9.7	1	2	55677	8.2	1	UTP--glucose-1-phosphate uridylyltransferase (UDPGP) (UGPase)
B0YJC4	63.6	8	20	49653	5.2	20	Vimentin variant 3;
Q9H0D2_ISOFORM_2	8.5	1	1	116894	8.5	1	Zinc finger protein 541 [ISOFORM 2]
O60844_CHAIN_0	14.8	2	20	16661	9.6	1	Zymogen granule membrane protein 16 (Zymogen granule prote

Supplementary Information 10: Analysis of pathways representation of soluble fraction

Name of this Event	Un-adjusted probability of seeing N or more genes in this Event by chance	Number of identifiers dataset which map to this Event	Total number of identifiers involved in this Event
Platelet degranulation	1.20E-08	10	78
Response to elevated platelet cytosolic Ca2+	2.22E-08	10	83
Glucose metabolism	3.66E-07	8	62
Uptake of Carbon Dioxide and Release of Oxygen by Erythrocytes	1.00E-06	4	8
Uptake of Oxygen and Release of Carbon Dioxide by Erythrocytes	1.00E-06	4	8
O2/CO2 exchange in erythrocytes	1.00E-06	4	8
Release of (inferred) platelet cytosolic components	5.44E-06	3	4
Glycolysis	1.05E-05	5	27
Metabolism	1.07E-05	33	1442
Activation of Chaperone Genes by ATF6-alpha	1.35E-05	3	5
Platelet activation, signaling and aggregation	1.56E-05	11	205
Sema3A PAK dependent Axon repulsion	1.84E-05	4	15
Gluconeogenesis	2.14E-05	5	31
Citric acid cycle (TCA cycle)	5.06E-05	4	19
Activation of Chaperones by ATF6-alpha	7.38E-05	3	8
Semaphorin interactions	8.85E-05	6	66

Protein identifiers covering the list of all identified proteins were entered into Reactome Instance Browser (accessed 27.02.13). Pathways and subpathways with a P-value <10-4 are listed, along with information on proportion of pathway identified.

Supplementary Information 11: Protein interaction pathways in the soluble fraction

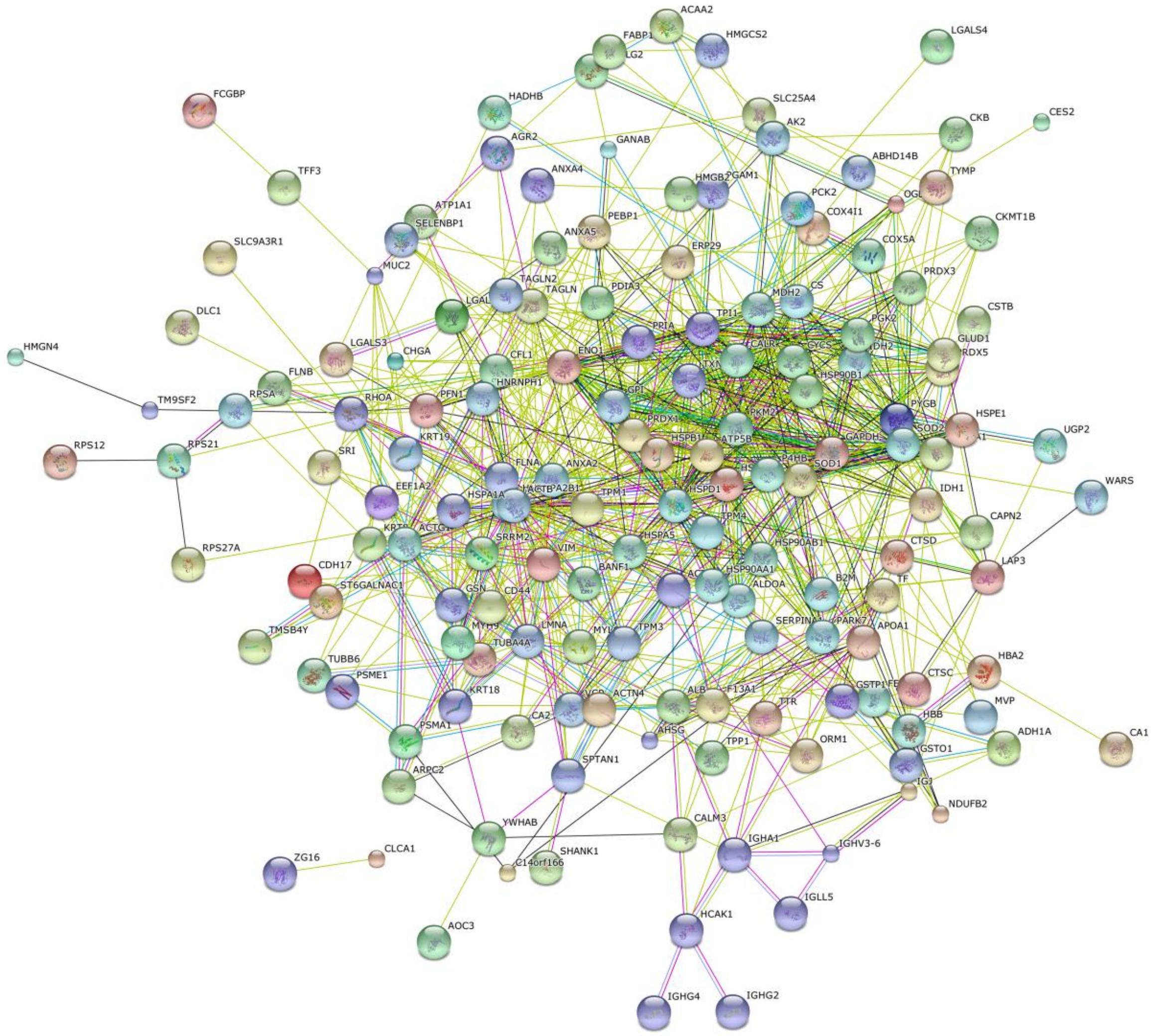

Part A. A protein interaction network was generated from the list of identified proteins in section 9 above. The network was generated in STRINGS version 9.0 (accession date 27.02.13). Orphan nodes are excluded from the representation. The above version includes information from text mining, represented by a green edge.

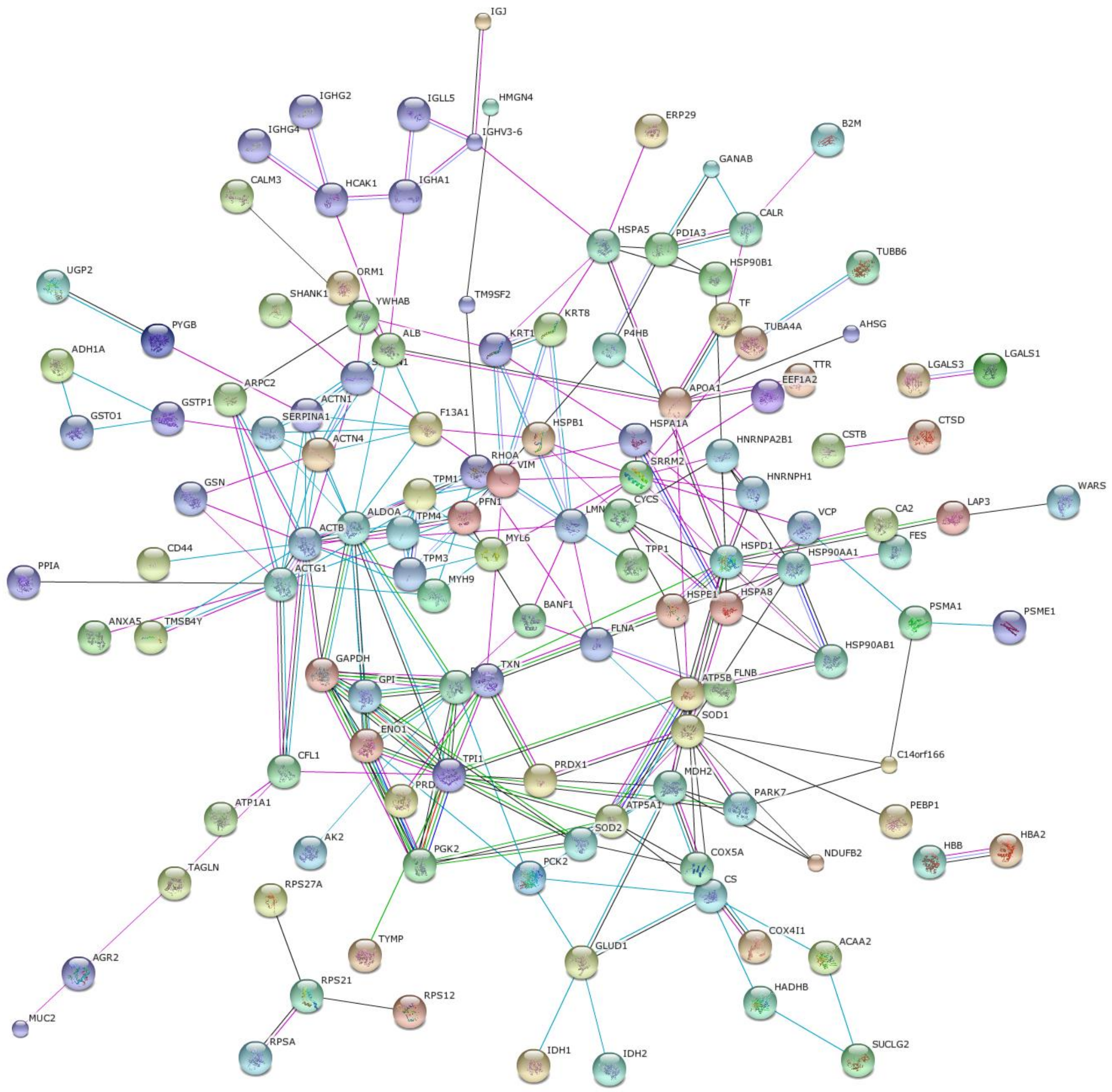

Part B. A protein interaction network was generated from the list of identified proteins in section 9 above. The network was generated in STRINGS version 9.0 (accession date 27.02.13). Orphan nodes are excluded from the representation. The above version excludes information from text mining.

Adenoma, mid sigmoid, high butyrate vs Normal, mid sigmoid, high butyrate						
Accession nu	Protein	# unique pe	% coverage	# peptides for quant	115:113	p-value
Q8WUW7	Pyruvate kinase	4	14	6	3.01471	0.00885
B4DP56	cDNA FLJ52237, highly similar to Creatine kinase B-type	4	12	8	1.41203	0.048608
P35579	Myosin-9	6	4	6	1.41013	0.019363
C9JEY0	Putative uncharacterized protein HADHB	2	12	3	1.32458	0.012525
P00918	Carbonic anhydrase 2	3	13	4	1.28892	0.021014
P69905	Hemoglobin subunit alpha	4	30	30	1.20429	0.021509
P68871	LVV-hemorphin-7	8	59	80	1.10931	0.017686
P06733	Alpha-enolase	8	16	18	0.83609	0.020562
Q9UNM1	Chaperonin 10-related protein	4	31	21	0.81407	0.012273
P62937	Peptidyl-prolyl cis-trans isomerase A	7	38	11	0.75710	0.006668
B7Z2X4	cDNA FLJ53327, highly similar to Gelsolin	4	5	5	0.69201	0.030893
P09493	Tropomyosin alpha-1 chain	6	17	2	0.41615	0.01481
Adenoma, mid sigmoid, low butyrate vs Normal, mid sigmoid, low butyrate						
Accession nu	Protein	# unique pe	% coverage	# peptides for quant	115:113	p-value
C9JEY0	Putative uncharacterized protein HADHB	2	12	3	1.89611	0.007802
Q14400	GLUD1 protein	2	11	2	1.84097	0.013709
A8MW49	Putative uncharacterized protein FABP1	4	28	5	1.71105	0.006486
P08727	Keratin, type I cytoskeletal 19	8	20	17	1.30970	0.019336
O60744	Thioredoxin delta 3	3	37	9	1.28267	0.009616
P00918	Carbonic anhydrase 2	3	13	4	1.27860	0.024788
P30044	Peroxiredoxin-5, mitochondrial	2	10	11	1.27458	0.019269
Q9UNM1	Chaperonin 10-related protein	4	31	21	1.26111	0.00752
P60174	Triosephosphate isomerase	4	19	13	1.23517	0.036699
B3KQT9	cDNA PSEC0175 fis, highly similar to Protein disulfide-isomerase A3	5	10	6	1.19445	0.044497
P56470	Galectin-4	6	16	18	1.18915	0.033808
P68871	LVV-hemorphin-7	8	59	80	0.85104	0.002871
P02768	Serum albumin	28	37	121	0.74812	4.11E-08
P00738	Haptoglobin beta chain	5	10	5	0.54273	0.037146
Q53GC9	Transgelin variant	4	21	6	0.54153	0.02818

Comparisons of the effect adenomagenesis, controlling for butyrate level.

Numbers of peptides used in each identification and for protein quantification are shown. Ratio between reporter ions (a proxy measure of fold-change) is show in column 6. Proteins identified as altered with a p-value <0.05 are shown in this table. Protein differences were identified in subjects with both high and low butyrate levels in stool.

Low butyrate

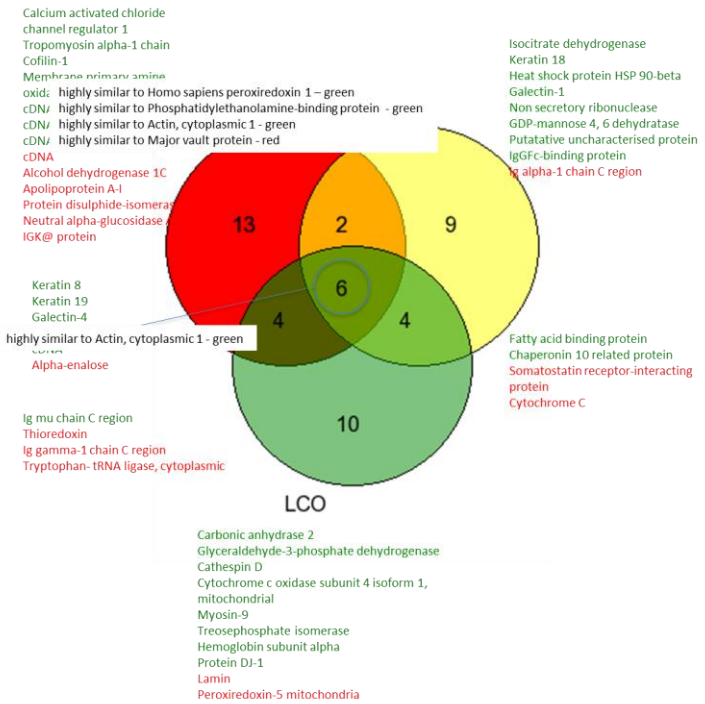

High butyrate

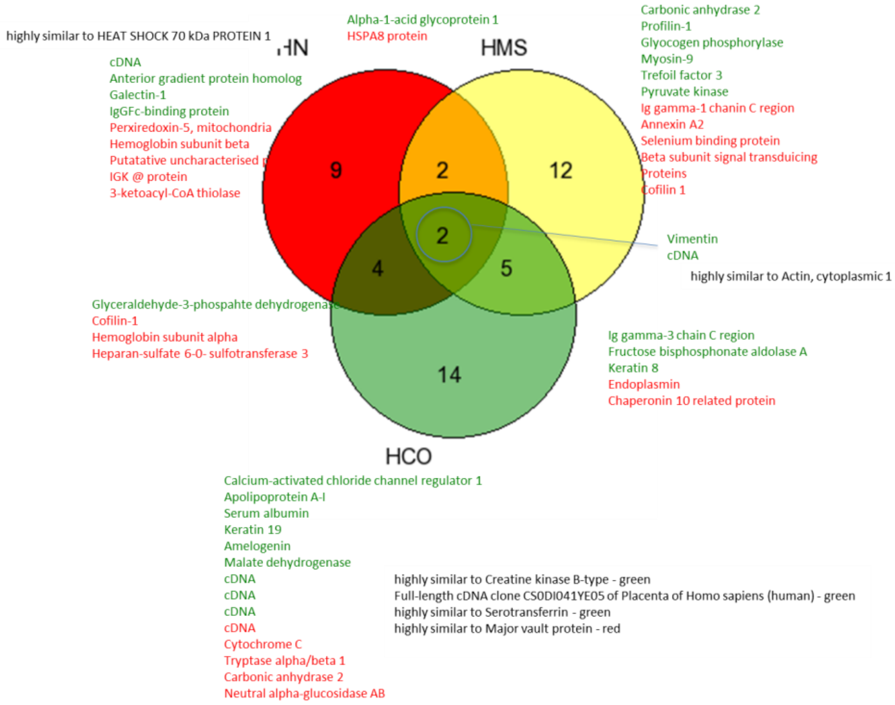

Normal, mid sigmoid, low butyrate vs Normal, mid sigmoid, high butyrate						
Accession nu	*Protein*	*# unique peptide*	*% coverage*	*# peptides for quant*	*115:113*	*p-value*
P09493	Tropomyosin alpha-1 chain	6	17	2	1.8	0.014757
A8K7I4	Calcium-activated chloride channel regulator 1	8	9	14	1.7	0.001841
O43707	Alpha-actinin-4	7	8	5	1.4	0.017473
P04080	Cystatin-B	3	34	8	1.3	0.021841
P68871	LVV-hemorphin-7	8	59	80	1.2	1.47E-05
B4E335	cDNA FLJ52842, highly similar to Actin, cytoplasmic 1	12	36	44	1.2	0.007378
P02768	Serum albumin	28	37	121	1.2	0.00022
Q9UNM1	Chaperonin 10-related protein	4	31	21	0.8	0.024277
P04406	Glyceraldehyde-3-phosphate dehydrogenase	8	24	13	0.8	0.042782
P62937	Peptidyl-prolyl cis-trans isomerase A	7	38	11	0.7	0.019938
P68104	Elongation factor 1-alpha 1	6	15	9	0.7	0.03489
A8MW49	Putative uncharacterized protein FABP1	4	28	5	0.6	0.002161
B4DW73	cDNA FLJ50710, highly similar to Phosphoenolpyruvate carboxyk	3	5	3	0.6	0.003643
P11021	78 kDa glucose-regulated protein (GRP-78)	3	5	4	0.5	0.043503
Q14400	GLUD1 protein	2	11	2	0.3	0.020021
**Adenoma, contralateral, low butyrate vs Adenoma, contralateral, high butyrate**						
Accession nu	*Protein*	*# unique peptide*	*% coverage*	*# peptides for quant*	*115:113*	*p-value*
Q53GC9	Transgelin variant	4	21	6	2.0	0.008861
B3KSV9	cDNA FLJ37148 fis, highly similar to Homo sapiens Na+/H+ excha	2	7	2	1.5	0.040968
P07339	Cathepsin D heavy chain	2	5	5	1.4	0.026791
P60174	Triosephosphate isomerase	4	19	13	1.4	0.006505
P01876	Ig alpha-1 chain C region	6	17	22	1.3	0.003091
P68871	LVV-hemorphin-7	8	59	80	1.2	3.37E-05
Q9UNM1	Chaperonin 10-related protein	4	31	21	1.1	0.034147
P02768	Serum albumin	28	37	121	0.8	0.000383
B3KPS3	cDNA FLJ32131 fis, clone PEBLM2000267, highly similar to Tubuli	4	13	13	0.8	0.031776
P05787	Keratin, type II cytoskeletal 8	13	25	25	0.8	0.018792
P02647	Apolipoprotein A-I(1-242)	7	26	17	0.7	0.012613
P08727	Keratin, type I cytoskeletal 19	8	20	17	0.7	0.035073
P68104	Elongation factor 1-alpha 1	6	15	9	0.7	0.023343
Q13747	Alpha-1 antitrypsin	3	15	4	0.7	0.023864
B0YJC4	Vimentin variant 3	8	20	20	0.7	0.01397
P07900	Heat shock protein HSP 90-alpha	6	7	6	0.6	0.017197
P09493	Tropomyosin alpha-1 chain	6	17	2	0.4	0.003849

Comparisons of the effect butyrate on the mucosal proteome, controlling for diagnosis and proximity to adenoma. Numbers of peptides used in each identification and for protein quantification are shown.Ratio between reporter ions (a proxy measure of fold-change) is show in column 6. Proteins identified as altered with a p-value <0.05 are shown in this table. Protein differences were identified in subjects with both high and low butyrate levels in stool.

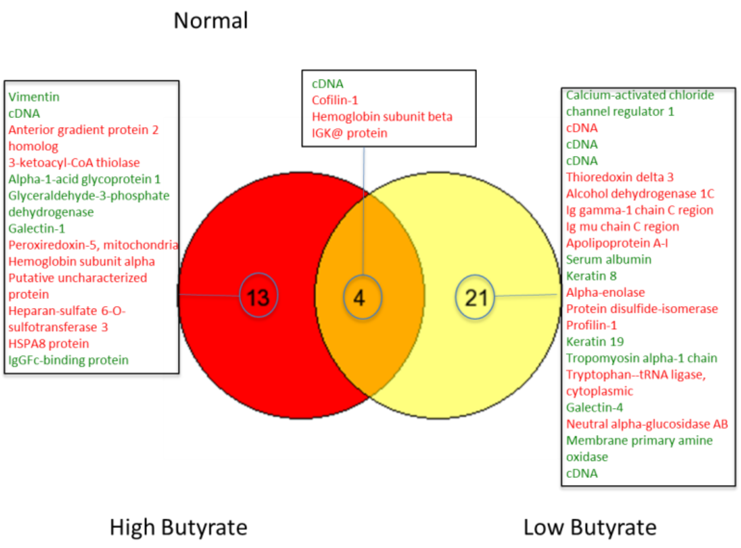
Normal
Vimentin
cDNA
Anterior gradient protein 2 homolog
3-ketoacyl-CoA thiolase
Alpha-1-acid glycoprotein 1
Glyceraldehyde-3-phosphate dehydrogenase
Galectin-1
Peroxiredoxin-5, mitochondria
Hemoglobin subunit alpha
Putative uncharacterized protein
Heparan-sulfate 6-O-sulfotransferase 3
HSPA8 protein
IgGFc-binding protein
cDNA
Cofilin-1
Hemoglobin subunit beta
IGK@ protein
Calcium-activated chloride channel regulator 1
cDNA
cDNA
cDNA
Thioredoxin delta 3
Alcohol dehydrogenase 1C
Ig gamma-1 chain C region
Ig mu chain C region
Apolipoprotein A-I
Serum albumin
Keratin 8
Alpha-enolase
Protein disulfide-isomerase
Profilin-1
Keratin 19
Tropomyosin alpha-1 chain
Tryptophan--tRNA ligase, cytoplasmic
Galectin-4
Neutral alpha-glucosidase AB
Membrane primary amine oxidase
cDNA
13
4
21
High Butyrate
Low Butyrate

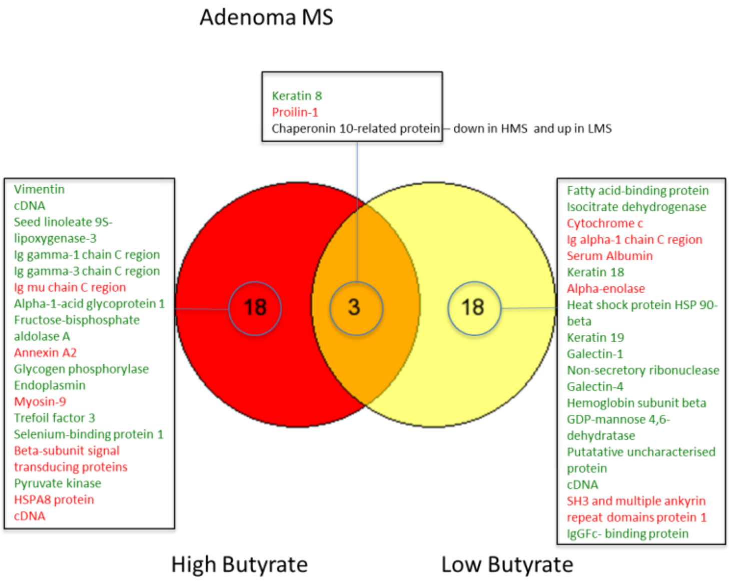
Adenoma MS
Keratin 8
Proilin-1
Chaperonin 10-related protein
down in HMS and up in LMS
Vimentin
cDNA
Seed linoleate 9S-lipoxygenase-3
Ig gamma-1 chain C region
Ig gamma-3 chain C region
Ig mu chain C region
Alpha-1-acid glycoprotein 1
Fructose-bisphosphate aldolase A
Annexin A2
Glycogen phosphorylase
Endoplasmin
Myosin-9
Trefoil factor 3
Selenium-binding protein 1
Beta-subunit signal transducing proteins
Pyruvate kinase
HSPA8 protein
cDNA
Fatty acid-binding protein
Isocitrate dehydrogenase
Cytochrome c
Ig alpha-1 chain C region
Serum Albumin
Keratin 18
Alpha-enolase
Heat shock protein HSP 90-beta
Keratin 19
Galectin-1
Non-secretory ribonuclease
Galectin-4
Hemoglobin subunit beta
GDP-mannose 4,6-dehydratase
Putatative uncharacterised protein
cDNA
SH3 and multiple ankyrin repeat domains protein 1
IgGFc- binding protein
18
3
18
High Butyrate
Low Butyrate

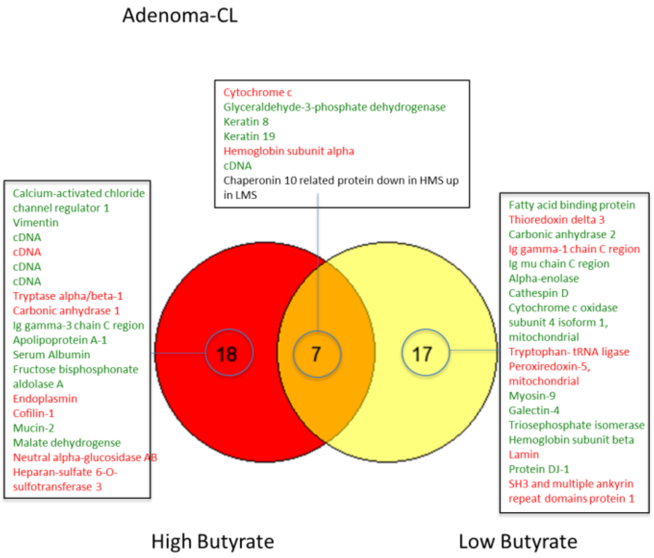
Adenoma-CL
Cytochrome c
Glyceraldehyde-3-phosphate dehydrogenase
Keratin 8
Keratin 19
Hemoglobin subunit alpha
cDNA
Chaperonin 10 related protein down in HMS up in LMS
Calcium-activated chloride channel regulator 1
Vimentin
cDNA
cDNA
cDNA
cDNA
Tryptase alpha/beta-1
Carbonic anhydrase 1
Ig gamma-3 chain C region
Apolipoprotein A-1
Serum Albumin
Fructose bisphosphonate aldolase A
Endoplasmin
Cofilin-1
Mucin-2
Malate dehydrogense
Neutral alpha-glucosidase AB
Heparan-sulfate 6-O-sulfotransferase 3
Fatty acid binding protein
Thioredoxin delta 3
Carbonic anhydrase 2
Ig gamma-1 chain C region
Ig mu chain C region
Alpha-enolase
Cathespin D
Cytochrome c oxidase subunit 4 isoform 1, mitochondrial
Tryptophan- tRNA ligase
Peroxiredoxin-5, mitochondrial
Myosin-9
Galectin-4
Triosephosphate isomerase
Hemoglobin subunit beta
Lamin
Protein DJ-1
SH3 and multiple ankyrin repeat domains protein 1
18
7
17
High Butyrate
Low Butyrate

Supplementary Information 12: Western Immunoblot Orthogonal Validation of Targets altered in response to butyrate

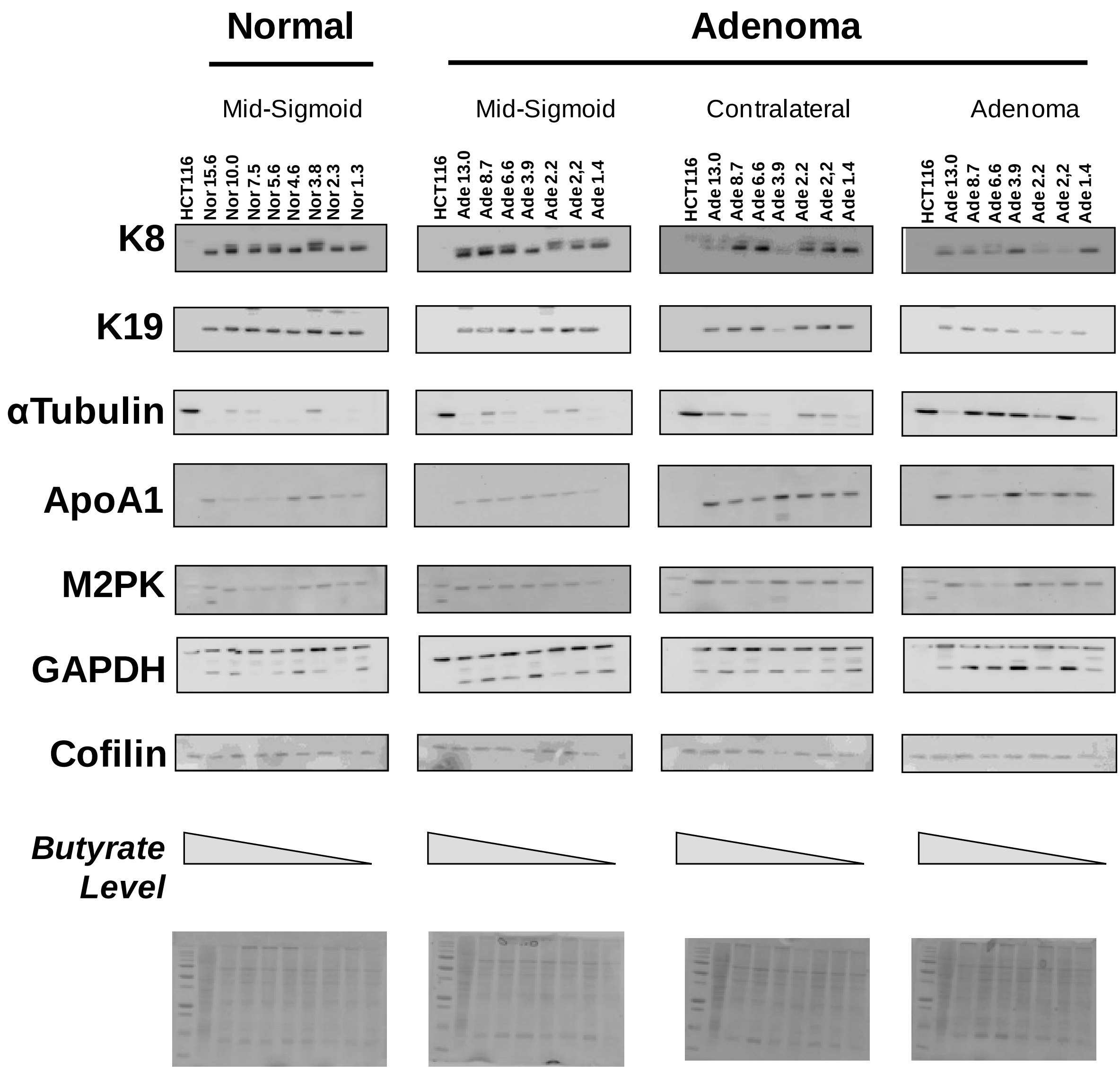

A subset of proteins found to be most altered in the iTRAQ analysis were chosen for validation by immunoblotting as an independent methodology. The rationale for choice included degree of change, axis of change (by butyrate or by disease progression), biological/clinical relevance to colon carcinogenesis and, pragmatically, availability of a commercial antibody. Seven proteins were selected in all: keratin 8, keratin 19, α-tubulin, ApoA1, M2PK, GAPDH and cofilin. For analysis of the effect of butyrate on expression of these proteins, the full set of samples from the study were grouped primarily by disease status and secondarily by sample position into normal (mid-sigmoid), adenoma (mid-sigmoid sample), adenoma (contralateral sample), adenoma (adenoma sample) and then ranked by butyrate status of the pools (as shown in Fig 1C). Western blots of each ranked group

were immunoprobed with the antibodies indicated. All data are shown in Figure 3. As many proteins' expression are labile in the presence of butyrate, including commonly used gel loading controls such as tubulin, coomassie blue-stained gels have been used to show that the samples contain similar amounts of protein.

Trends can be seen in the samples for a number of proteins, although there are differences between groups. For keratins 8 and 19 there is a decrease in expression associated with decreasing butyrate level that is marked and consistent in the mid-sigmoid samples of the adenoma group, but less pronounced or absent from the normal group. The trend is consistent with both proteins. However the trend is flattened in the adenoma and contralateral samples, implying there may be an over-riding field effect. There is a trend towards increased expression of ApoA1 and M2PK with decreasing butyrate in the samples from normal subjects, which is not matched in samples from any of the adenoma groups. GAPDH and cofilin did not appear to be influenced by butyrate status in any group.

Supplementary Information 13: Western Immunoblot Orthogonal Validation of Targets altered in response to lesional proximity

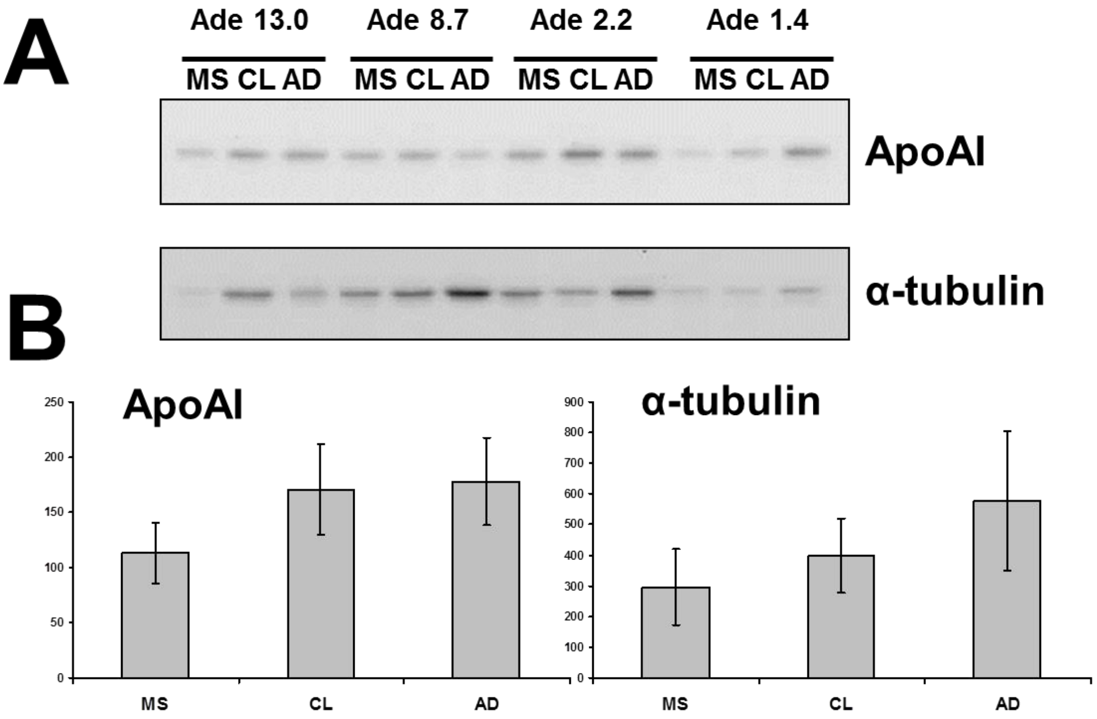

Supplementary Information 14: Comparison of subgroups in the normal population for keratin endpoints assessed by IHC

Surface intensity P=0.33

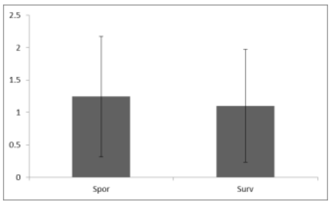

Crypt intensity P=0.47

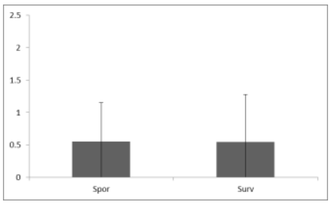

Crypt Depth P=0.28

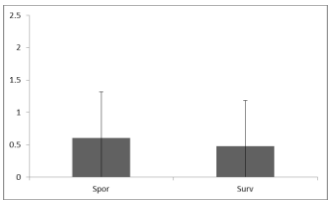

Subjects in the Normal group were recruited from both adenoma surveillance clinics and new cases, and therefore represent a mix of individuals for which a sub-population may continue to exhibit the changes in keratin expression observed associated with the presence of adenoma. Retrospective data from subjects where available as to previous history allowed the division into sporadic (Spor, n=9) and surveillance (Surv, n=9) subgroups. The mid-sigmoid K8 scores for each group were compared (above panels). There were no significant differences or trends between these groups.

Supplementary Information 15: 2DGE Analysis of acetylome

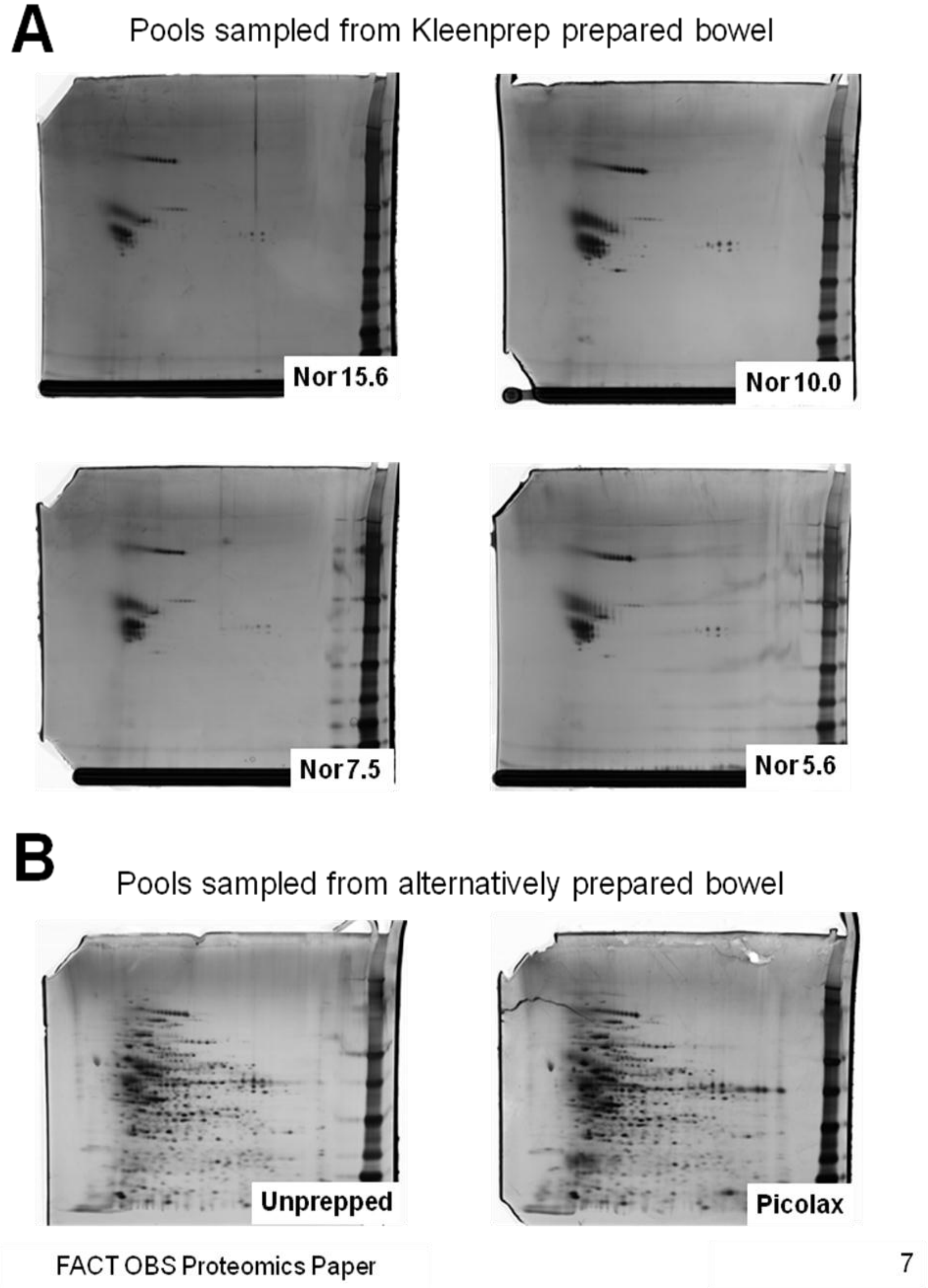

Panel A shows 2d gel images from four pools from subjects with normal bowel pathology and mean faecal butyrate of 15.5, 10.0, 7.5 and 5.6 mM respectively. The numbers of protein spots are extremely

low by comparison with sample from Pico-lax-prepared bowel and from unprepared bowel (Panel B), which are broadly similar in distribution and intensity.

Following fractionation of samples, the IP eluate, enriched for acetyl proteins, was analysed by 2DGE. One eluate (representing a pool of 4 subjects with similar faecal butyrate) was run on each 2d gel. Pools included the higher and lower butyrate range from normal, and from the multiple positions of subjects with adenoma. Two additional samples were included as a result of the scope of the study– a pool from biopsies extracted after Picolax, and a pool from biopsies taken intraoperatively during cancer resection (which was undertaken without bowel prep). The numbers of protein spots appearing on the gels from subjects prepared with Kleanprep, irrespective of faecal butyrate concentration, was extremely low (Panel A). In order to distinguish between a technical failure and genuine alteration protein acetylation, samples from unprepared and Picolax-prepared bowel were compared (Panel B). Both showed a spot distribution comparable or slightly enriched from that achieved in method work-up using cell line material (LJC & BMC unpublished). As the majority of samples acquired for this study (and indeed in general GI clinics, are derived from PEG-based bowel prep) this analytical arm was suspended.
